# GlcNac produced by the gut microbiome enhances host influenza resistance by modulating NK cells

**DOI:** 10.1080/19490976.2023.2271620

**Published:** 2023-11-12

**Authors:** Xiaotong Hu, Xiaolu Sun, Ya Zhao, Changjie Iv, Xiaomei Sun, Meilin Jin, Qiang Zhang

**Affiliations:** a State Key Laboratory of Agricultural Microbiology, Huazhong Agricultural University, Wuhan, China; b College of Veterinary Medicine, Huazhong Agricultural University, Wuhan, China; c Key Laboratory of Development of Veterinary Diagnostic Products, Ministry of Agriculture, Wuhan, China; d Emerging Disease Research Center, Keqian Institute of Biology, Keqian Biological Co. Ltd, Wuhan, China; e College of Biomedicine and Health, Huazhong Agricultural University and Hubei jiangxia Laboratory, Wuhan, China

**Keywords:** Influenza, gut microbiome, heterogeneity, influenza virus resistance, N-acetyl-D-glucosamine, prognosis predictor

## Abstract

Microbiota are known to modulate the host response to influenza infection, but the mechanisms remain largely unknown. Gut metabolites are the key mediators through which gut microbes play anti-influenza effect. Transferring fecal metabolites from mice with high influenza resistance into antibiotic-treated recipient mice conferred resistance to influenza infections. By comparing the metabolites of different individuals with high or low influenza resistance, we identified and validated N-acetyl-D-glucosamine (GlcNAc) and adenosine showed strong positive correlations with influenza resistance and exerted anti-influenza effects in vivo or in vitro, respectively. Especially, GlcNAc mediated the anti-influenza effect by increasing the proportion and activity of NK cells. Several gut microbes, including *Clostridium sp*., *Phocaeicola sartorii*, and *Akkermansia muciniphila*, were positively correlated with influenza resistance, and can upregulate the level of GlcNAc in the mouse gut by exogenous supplementation. Subsequent studies confirmed that administering a combination of the three bacteria to mice via gavage resulted in similar modulation of NK cell responses as observed with GlcNAc. This study demonstrates that gut microbe-produced GlcNAc protects the host against influenza by regulating NK cells, facilitating the elucidation of the action mechanism of gut microbes mediating host influenza resistance.

## Introduction

Gut microbiota is essential to the maintenance of normal life activities.^1–4^ Gut microbiota may modulate host immune responses by integrating environmental signals,^5–8^ profoundly affecting the development of cancer,^9–11^ metabolic diseases^12,13^ and pathogenic infections.^14–16^ Changes in gut microbiota can not only affect local cell function and immune response but also play an important role in the pathogenesis of respiratory diseases.^17^ However, the mechanisms by which gut microbiota protect distal organs from infection have not been fully elucidated.

The influenza virus belongs to the Orthomyxoviridae family^18^ that is responsible for serious acute communicable respiratory illnesses. In the past few decades, the influenza pandemic has seriously threatened public health at global scale. Four major influenza pandemics occurred in recent human history: H_1_N_1_ in 1918, H_2_N_2_ in 1957, H_3_N_2_ in 1968,^19^ and swine-origin H_1_N_1_ in 2009.^20^ The 1918 influenza pandemic is considered a typical example of poor disease prevention and control management, and led to at least 40 million deaths.^21^ According to the World Health Organization, seasonal influenza viruses are responsible for 3–5 million cases of severe illness and 290,000–650,000 deaths worldwide each year.^22^ New influenza strains appear by genome segment reassortment between existing influenza viruses of human or animal origin or spontaneous mutations that occasionally cross the species barrier.^19,23^ For example, in March 2013, H_7_N_9_ HPAIV first emerged in China and caused 1568 infections and 616 human deaths,^24^ inflicting a large toll on the poultry industry. The infection of influenza virus to host is highly influenced by the activity of gut microbiota, with the disruption or removal of specific microbiota resulting in adverse outcomes.^24,25^ Several mechanisms have been elucidated in which gut microbiota enhance host resistance, including through the activation of toll-like receptors^24^ or regulation of the IFN-driven antiviral state in lung stroma.^26^ Microbial metabolites are known to modulate various important systemic phenotypes,^8,27,28^ and several studies have demonstrated that gut microbiota participate in many diseases through metabolites, including influenza infection.^29,30^ However, many details remain to be studied.

Heterogeneity in the host response to infection results in different degrees of pathological phenotypes. The immune system deals with infection by two distinct strategies: virus resistance and disease tolerance. In this study, we investigated how gut microbiota affect host resistance to influenza by screening functional gut metabolites between mice with different degrees of influenza resistance using metabolomic analysis. We identified eight metabolites that were positively correlated with influenza resistance and described the anti-influenza activities of N-acetyl-D-glucosamine (GlcNAc) and adenosine in vivo and in vitro. We investigated the underlying anti-influenza mechanism of GlcNAc and identified the gut microbes associated with its generation. This study provides additional insight into the anti-influenza effects of gut microbiota and provides an empirical basis for the development of novel anti-influenza drugs.

## Results

### Gut metabolites from high anti-influenza mice potentiated the influenza resistance in recipient mice

**Figure 1.**
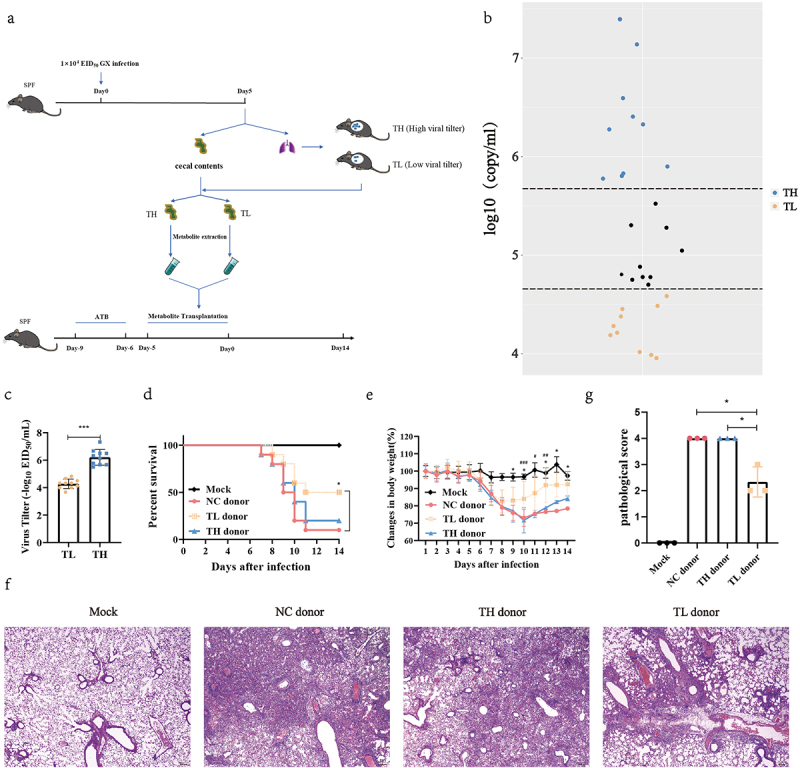
Heterogeneity of mouse influenza resistance is closely associated with gut metabolites.

To evaluate whether the heterogeneity of influenza infection is associated with gut microbial metabolites, we designed an experiment as described in Figure 1a. We found that virus content varied significantly between individual mice when mice were intranasally inoculated with the same dose of influenza virus (Figure 1b). The interindividual differences observed in normal mice became less significant when the mice were treated with antibiotics (Fig. S1). These results suggest that mice exhibited different responses to infection under the same conditions and gut microbes play a crucial role in the diverse host responses to infection. Next, mice were sorted according to its viral load, and the top 1/3 of the viral load was divided into the TH group (low influenza virus resistance), while the bottom 1/3 of the viral load was divided into the TL group (high influenza virus resistance) (Figure 1c). The gut metabolite extracts from TH, TL, and NC group mice, as described previously.^31^ These water-soluble intestinal extracts, which mainly contained gut microbial metabolites, were orally administered to antibiotic-pretreated recipient mice for 5 d before the recipient mice were infected with influenza virus. Compared with TH and NC donor groups, the gut microbial metabolites from the TL group increased the survival rate of the recipient mice by 30% or 40% (*P* = .046) (Figure 1d) and promoted weight loss (*P* < .05) (Figure 1e). There was no difference in survival or weight change between the TH and NC donor groups (Figure 1d,e). In addition, the histopathology of mice after 7 d of infection showed that inflammatory cell infiltration, alveolar atrophy, and fibrosis in the lungs of TL group mice were alleviated (Figure 1f), and the pathological scores were significantly reduced (*P* = .016) (Figure 1g). This suggests that gut metabolite variation influences host resistance to influenza virus infection, and that the fecal microbiota of mice in the TL group likely contain specific gut metabolites that lend resistance to infection.

(a)Experimental setup for IMT experiments. Cecal contents were collected 5 d after GX infection (*n* = 30); 20 mice were divided into high (*n* = 10, TH) and low virus titer (*n* = 10, TL) groups based on the level of viral load in the lungs. The water-soluble metabolites were extracted and transplanted to SPF mice pretreated with an antibiotic solution for 3 d. After 5 d, the mice were infected with 1 × 10^4^ EID_50_ GX. (b) Viral RNA transcript counts in the lungs of infected SPF mice at 5 dpi. (c) Viral titer in the lungs of infected SPF mice at 5 dpi. (d) survival and (e) weight change curves for the TH donor, TL donor, and NC donor (NC, mock infection using PBS) group after metabolite transplantations (*n* = 10 per group). (f) Representative images of an H&E staining mouse lung. In another repeat metabolite transplantation experiment, the mice were sacrificed at 7 dpi and the lungs were collected. Survival was assessed with a log-rank (Mantel-Cox) test. (g) Blinded sections were assessed to determine the level of pathological severity. In order to evaluate the overall histological changes, lung tissue sections were scored according to the criteria specified in the panel. The scoring system used was as follows: 0, no pathological change; 1, affected area (≤10%); 2, affected area (<50%, >10%); 3, affected area (≥50%). Weight changes were assessed using a two-way ANOVA. *P < .05, ***P <.001 (TL donor vs NC donor); ^#^*P* < .05, ^##^*P* < .01, and ^##^*P* < .001 (TL donor vs TH donor). Error bars show the mean ± SD for each treatment. All experiments were performed at least twice under similar conditions.

### The upregulation of eight gut metabolites were correlated to high resistance

**Figure 2.**
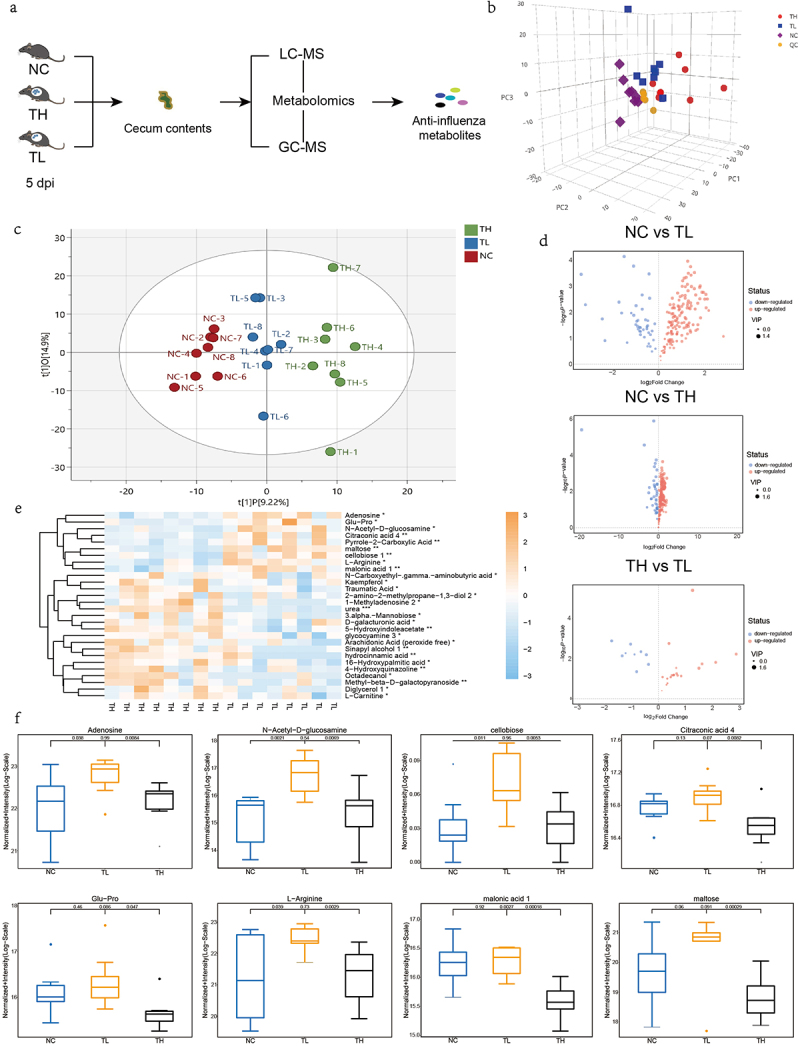
The influenza resistance of infected mice is associated with the enrichment of several gut metabolites.

To identify the metabolites that influenced resistance to the influenza virus, we reproduced the infection model as described above (Figure 1a). Mice exhibited significant inter-individual heterogeneity in lung virus load after infection with the same dose of influenza virus at 5 d after infection (Fig. S1a, Table. S5). For more comprehensive screening, liquid chromatography-mass spectrometry (LC-MS) and gas chromatography-mass spectrometry (GC – MS) were used for untargeted metabolomics (Figure 2a). The principal component analysis (PCA) data revealed that all samples were within the 95% confidence interval (Figure 2b). Using orthogonal projections to latent structures-discriminant analysis (OPLS-DA, tested for good fit), we found significant differences between the three groups (Figure 2c and S2b). Based on a multivariate analysis – variable importance in projection (VIP) – we observed that the levels of a large number of metabolites were altered after infection compared with the NC group (*P* < .05) (Figure 2d). Moreover, there were significant differences in the levels of some metabolites between the TH and TL groups, though the differential metabolites between these groups were far fewer than those between the TH and NC groups. According to the Euclidean distance matrix, which clustered differentially expressed metabolites using a complete chain method, 189 metabolites differed significantly in abundance between the TH and NC groups (*P* < .05) (Fig. S3a), while 182 metabolites differed significantly between the TL and NC groups (*P* < .05) (Fig. S3b). However, only 28 differentially expressed metabolites were identified between the TH and TL groups (*P* < .05) (Figure 2e, Table. S6). The comparative analysis has the advantage of eliminating interference from irrelevant metabolic factors, making it more conducive to screening potential anti-influenza metabolites. We assessed the relationship between the relative abundance of the 28 differential metabolites among the three groups to screen metabolites with anti-influenza activity. We found that eight metabolites were significantly upregulated in TL group compared to the TH group (*P* < .05). Additionally, these metabolites were found to be significantly higher than those in the NC group, or at the very least, maintained at similar levels as the NC group (Figure 2f). By analyzing the correlation between the relative abundance of eight metabolites and viral load, we found that the levels of adenosine (*P* = .025), GlcNAc (*P* =.032), Glu-Pro (*P* = .029), and maltose (*P* = .020) were negatively correlated with lung viral load (Fig. S4, Table. S7). Therefore, we speculated that these metabolites participated in host resistance to influenza virus infection.

(a) Protocols for Metabolome Sequencing. (b) The PCA of the sample in NC, TH, TL, and QC group (c) OPLS-DA showing differences in metabolite composition between the TH, TL, and NC groups. (d) Volcano plot comparison of NC vs TH, NC vs TL, and TH vs TL. Each point in the volcano plot represents a metabolite, the abscissa represents the fold change of each substance in the group compared to each other (taking the logarithm with base 2), and the ordinate represents the P-value of the Student’s *t*-test (taking the pair with base 10). The size of the scatter point corresponds with the VIP value of the OPLS-DA model. Significantly upregulated and downregulated metabolites are presented in red and blue, respectively. (e) Hierarchical clustering analysis of TH vs TL. The abscissa represents different experimental groups, the ordinate represents the differential metabolites compared in the group, the color blocks represent the relative expression levels of the metabolites at the corresponding positions, yellow indicates high expression levels, and blue indicates low expression levels. (f) Comparison of the relative content of each metabolite between the TH, TL, and NC groups. Data are presented as a minimum, first quartile, median, third quartile, and maximum. *P < .05, **P < .001.

### GlcNAc in vivo and adenosine in vitro exerted anti-influenza effects

**Figure 3.**
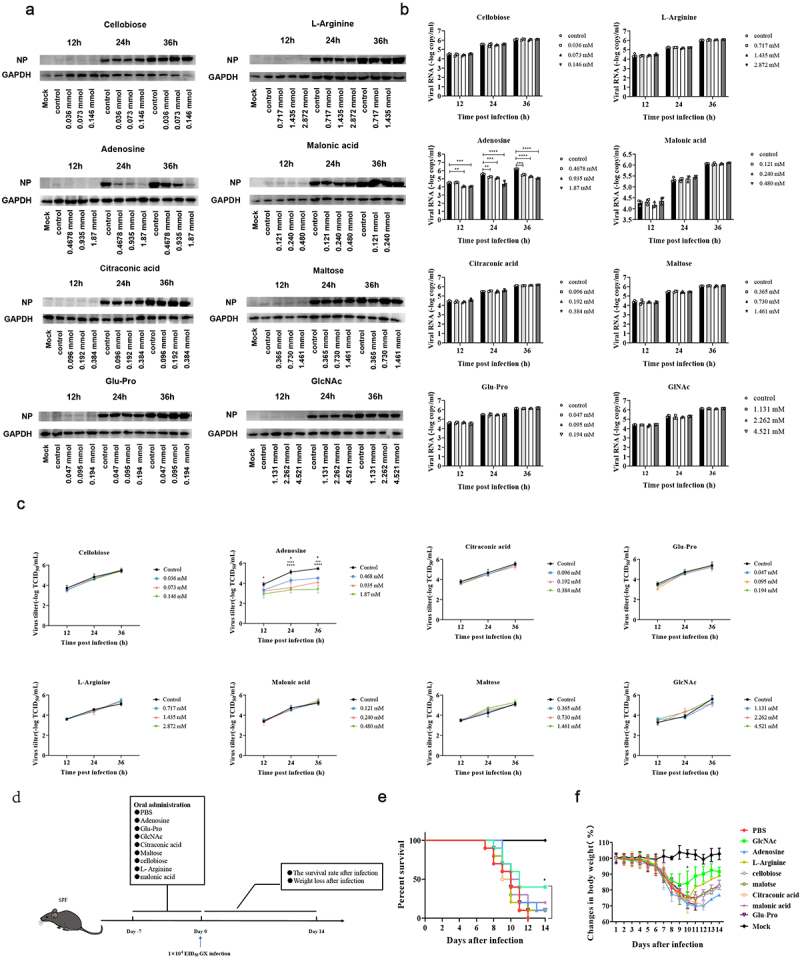
GlcNAc and adenosine exert anti-influenza effects in vivo and in vitro, respectively.

To verify our hypothesis, we evaluated the anti-influenza effects of these eight metabolites using in vitro and in vivo experiments. As these metabolites were isolated from the intestine, we chose Caco-2 cells as an in vitro model for infection given that they are highly permissive to GX influenza virus infection (Fig. S5a). We first evaluated the cytotoxicity of the eight metabolites and determined the optimal concentration (Fig. S5b). For each metabolite, the experiments were performed at three different concentrations in the nontoxic range. First, the mRNA level of NP was detected using RT-qPCR and the viral NP was measured by western blot. We found that NP expression was inhibited at all three concentrations of adenosine, in a dose-dependent manner (Figure 3a,b). Based on the virus titer estimated by TCID_50_ assay, adenosine treated cells suppressed virus proliferation (*P* < .05) compared with controls (Figure 3c). We also confirmed that the anti-influenza effect of adenosine persisted in Caco-2 cells infected with H5N6-GFP and (PR8) H_1_N_1_(*P* < .05) (Fig. S5c).

Caco-2 cells were pre-incubated with different concentrations of metabolites for 12 h, infected with the GX at an MOI of 0.1, and treated with the corresponding concentrations of metabolites. Cell supernatant samples were harvested 12, 24, and 36 h after infection. (a) The nucleoprotein (NP) level was measured in Caco-2 cells treated with different concentrations of metabolites. (b) The effect of metabolites on GX vRNA abundance at 12, 24, and 36 hpi. (c) The virus titer in the cell supernatant were detected by TCID_50_. (d) Workflow of the mouse experiment. SPF mice received daily oral administration for 1 week with GlcNAc, adenosine, L-arginine, cellobiose, maltose, citraconic acid, malonic acid or Glu-Pro, or the same volume of PBS (200 μL). All mice were intranasally inoculated with 1 × 10^4^ EID_50_ of H7N9 influenza virus (*n* = 10 per group). (e) Survival and (f) body weights of the remaining mice per group were monitored daily for 14 d. Statistics for vRNA abundance, virus titer, and weight changes were two-way ANOVA. Survival was assessed with the log-rank (Mantel-Cox) test. ^#^*P* < .05 (Control vs 0.468 mM); ^+++^*P* < .001, ^++++^*P* < .0001 (Control vs 0.935 mM); *P < .05, **P < .01; ***P < .001 (Control vs 1.87 mM). Error bars show the mean ± SD for each treatment

To evaluate the anti-influenza activities of these metabolites in vivo, we first tested their toxicity in mice. SPF mice were administered these metabolites at a 1/10 of the median lethal dose for 10 d, with no change in body weight (Fig. S5d) or health status compared with the PBS group. Recipient mice were administered PBS or metabolites by oral gavage for 1 week before intranasal inoculation with the influenza virus (Figure 3d). We found that oral administration of GlcNAc (500 µg/kg) increased the survival rate of recipient mice (*P* = .036) and reduced weight loss during infection at 9, 10 and 11 dpi (*P* = .033, *P* <  .001, *P* = .047) (Figure 3e, f). However, gavage with adenosine had no obvious effect.

**Figure 4.**
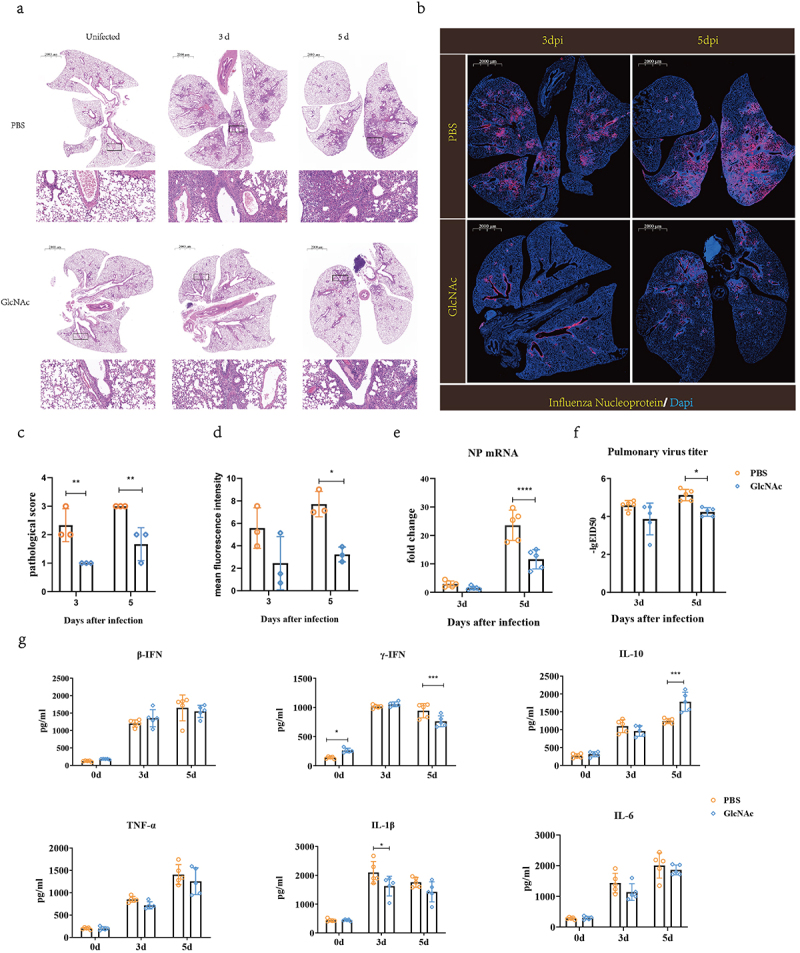
Oral administration of GlcNAc protects against H7N9 infection in recipient mice.

We evaluated the anti-influenza effects of GlcNAc according to lung virus titer, cytokine levels, and histological changes. Compared with the GlcNAc group at 3 and 5 dpi, severe lesions were found in the PBS group along with inflammatory cell infiltration, alveolar atrophy, and fibrosis (Figure 4a), and the pathological score was also significantly higher (*P* = .0079) (Figure 4c). Immunofluorescence staining of lung sections showed that the AIV NP protein occurred predominantly in the bronchi and bronchioles as well as in the bronchioalveolar-duct junction (BADJ) of the lungs. The fluorescence density of influenza virus nucleoprotein in lung tissue in GlcNAc group was also significantly lower than that in PBS group at 5 dpi. (*P* = .0193) (Figures 4b & 4d). At 3 and 5 dpi, pulmonary NP expression in the GlcNAc group was significantly less than that in the PBS group. Based on the mRNA levels of the viral NP determined by qRT-PCR (*P* < .001) (Figure 4e) and virus titers (*P* = .018) (Figure 4f), we confirmed that oral administration of GlcNAc inhibited the proliferation of the influenza virus in the lungs. According to the ELISA, the levels of pulmonary IFN-γ and IL-10 were significantly higher at 0 (*P* = .026) or 5 dpi (*P* < .001) in the GlcNAc group compared with the PBS group (Figure 4g). Conversely, IL-1β levels in the lungs were significantly lower at 3 dpi (*P* =.028). The IFN-β, TNF-α, and IL-6 levels did not vary between groups. When poly(I:C) was used as nasal drops to stimulate mice, we observed a significant decrease in TNF-α mRNA levels (*P* = .031) and a significant increase in IFN-γ mRNA levels (*P* = .047) in the GlcNAc group compared to the PBS group. However, there were no significant differences in IFN-β, IL-10, IL-1β, and IL-6 mRNA levels between the two groups (Fig. S6). These results suggested that GlcNAc can reduce some inflammatory factors after infection and exerts immunomodulatory effects, but acts independently of type I interferon production.

(a) Histological examination (H&E staining) and (b) immunohistochemistry of mice orally administrated 1000 mg/kg GlcNAc or PBS (200 μL) daily for 1 week and infected with H7N9 influenza virus GX. At 0, 3, and 5 dpi, three randomly selected mice per group were sacrificed. (c)Blinded sections were scored for levels of pathological severity. To evaluate comprehensive histological changes, lung tissue sections were scored based on criteria indicated in the panel. The following scoring system was used: 0, no pathological change; 1, affected area (≤10%); 2, affected area (<50%, >10%); 3, affected area (≥50%). (d) Quantitative analysis of the fluorescence intensity of NP in lung. The fluorescence intensity was calculated by the mean fluorescence intensity. In addition, 30 SPF mice were randomly divided into two groups and treated as described above. Lungs were collected from five randomly selected mice per group at 0, 3, and 5 dpi, and homogenized in PBS (1 mL/lung). (e) NP mRNA levels were measured by qRT-PCR. (f) Virus titers were measured using 10-d-old SPF embryonated chicken eggs. (g) Cytokine concentrations Weight changes, virus titers, and cytokine concentrations were assessed using a two-way ANOVA. *P < .05, ***P < .001, ****P < .0001. Error bars show the mean ± SD for each treatment.

### GlcNAc induced the proliferation of NK cells and enhanced the expression of IFN-γ and surface CD107a of NK cells in lung after influenza infection

**Figure 5.**
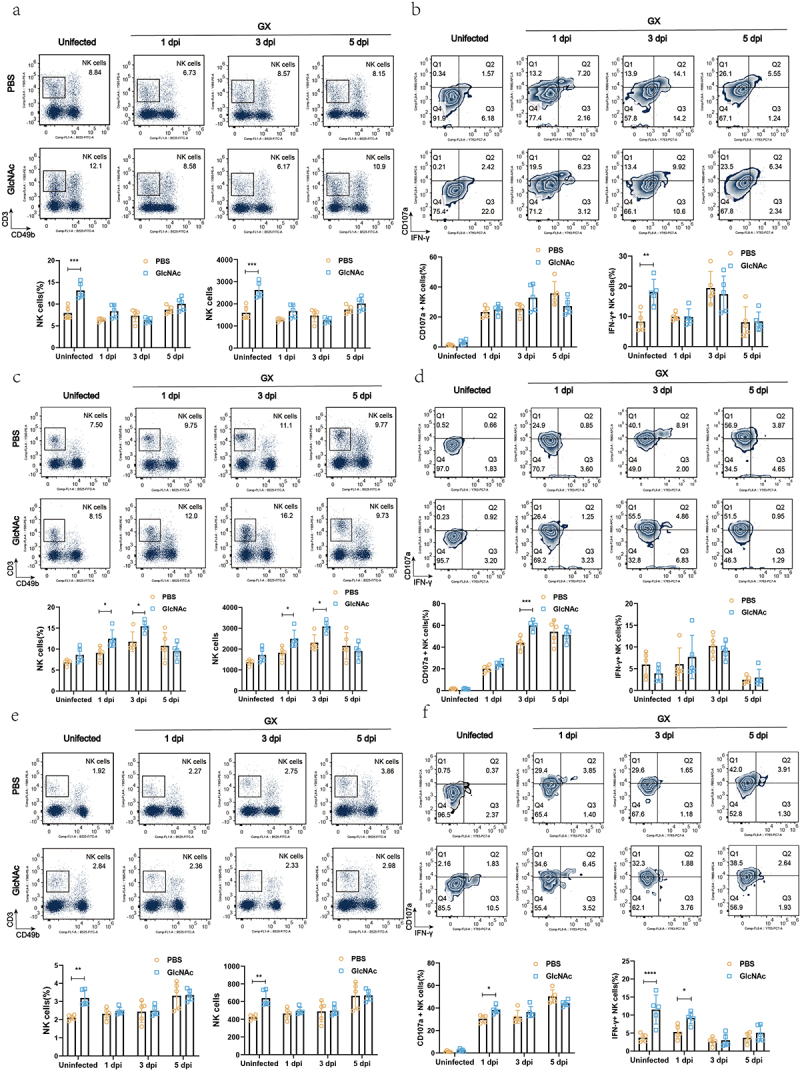
Oral administration of GlcNAc enhances NK cell responses before and after infection in mice.

To reveal the mechanism by which GlcNAc exerts anti-influenza effects, we analyzed the effects of GlcNAc treatment on immune cells, including NK cells, CD4+ T cells, and CD8+ T cells. We detected the proportion of CD4^+^ T, CD8^+^ T, and NK cells in the blood, lungs, and spleen of mice using flow cytometry before and after infection. There were no significant differences in the proportions of CD4^+^ and CD8^+^ T cells between the PBS and GlcNAc groups before or after infection, irrespective of sample type (Fig. S7 and S8). However, oral administration of GlcNAc significantly upregulated the proportion and number of NK cells in the blood (*P* < .001) and spleen (*P* < .01) of mice in the uninfected state (Figure 5a,e). A similar trend was observed in the lungs after treatment with GlcNAc, although this was not statistically significant (Figure 5c). The proportion of NK cells in the lungs of the GlcNAc group was markedly higher than that in the control group at 1 and 3dpi (*P* = .0417, *P* = .0229), though this difference disappeared at 5 dpi (Figure 5c). Compared with uninfected mice, the percentage of CD3-CD49b+NK cells in the lungs of the infected groups were higher at 1 dpi, continued to increase at 3 dpi, and then decreased at 5 dpi. In contrast, the NK cell percentage was dramatically reduced in peripheral blood at 1 dpi. This is consistent with a previous study showing that NK cells can migrate to infected organs through peripheral blood after viral infection^32^. The percentage of NK cells in the blood, spleen, and lungs of the two groups at different time points showed that oral administration of GlcNAc induced the proliferation of NK cells, especially in the lungs during early infection.

SPF mice received daily oral administration for 1 week with GlcNAc (500 mg/kg) or PBS (200 μL). All mice were intranasally inoculated with 1 × 104 EID50 of GX influenza virus. Lymphocytes from local draining mediastinal peripheral blood (a, b), lung (c, d), or spleen (e, f) were analyzed by flow cytometry. (a) Representative flow cytometry plots with gating strategy and summary graphs showing the proportion of NK cells in lymphocytes and total number of NK cell in peripheral blood post infection. (b) Frequency of IFN-γ and CD107a surface expression in gated CD3-CD49B+ NK cells. (c) As in (a), but showing the lymphocytes from the lung. (d) As in (b), but showing the NK cells from the lung. (e) As in (c), but showing the lymphocytes from the spleen. (d) As in (f), but showing the NK cells from the spleen. The proportion and number of NK cell and frequency of IFN-γ and CD107a surface expression in gated CD3-CD49b+. Flow cytometry data were assessed using a two-way ANOVA. *P < .05, **P < .01. Error bars show the mean ± SD for each treatment.

The expression of IFN-γ reflects the activation of NK cells, and surface expression of CD107a correlates with NK cell cytotoxicity.^33^ We found that IFN-γ+ NK cells between two groups showed a similar trend with corresponding NK cells in blood, i.e., the proportion and number of IFN-γ +NK cells were significantly increased in GlcNAc group compared with that in PBS group before infection (*P* = .0028, *P* < .0001) (Figure 5b and S9a) but no significant after infection. In the GlcNAc-treated group, IFN-γ+ NK cells in the spleen were significantly higher before infection (*P* < .0001) and at 1dpi (*P* = .0208) (Figure 5f and S9c). However, there was no significant difference in IFN-γ+ NK cells in the lungs between the two groups. Additionally, the GlcNAc group showed a significant increase in CD107a+ NK cells in the spleen at 1dpi (*P* = .0144) (Figure 5f and S9c) and in the lungs at 3dpi (*P* < .001) (Figure 5d and S9b), compared to the PBS group. Although there was no significant increase before infection. In the blood, the proportion of CD107a+ NK cells in the GlcNAc group were not significantly different from the PBS group, but the number of CD107a+ NK cells was significantly higher at 3dpi (*P* = .0394) (Fig. S9a). These results suggest that the oral administration of GlcNAc enhances NK cell responses before and after infection.

### GlcNAc exerted anti-influenza effects by regulating NK cells

**Figure 6.**
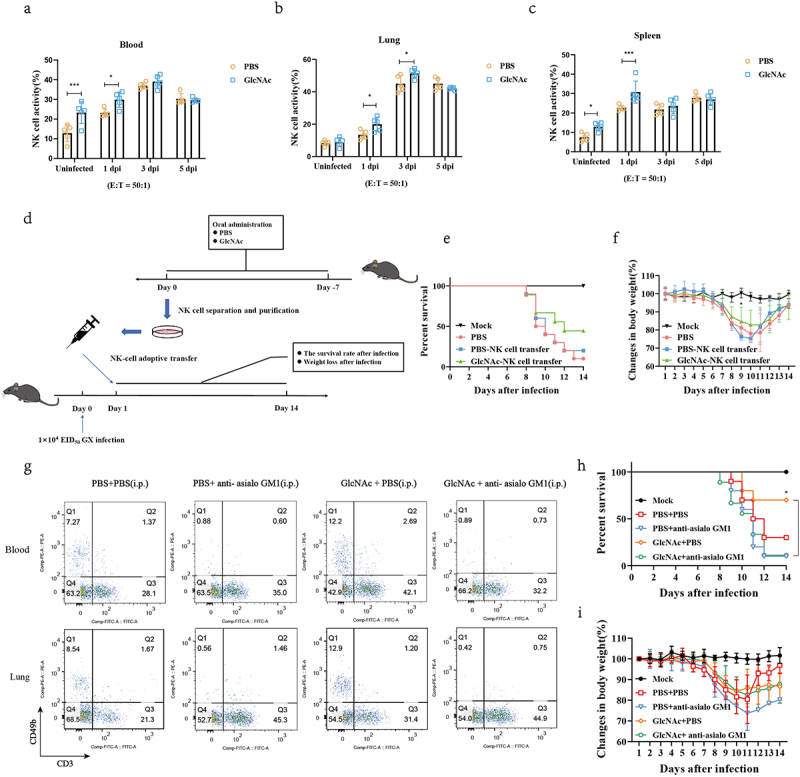
GlcNAc improves host defense against H7N9 infection by regulating NK cells.

To verify whether GlcNAc affects the killing activity of NK cells, we purified NK cells from PBS- or GlcNAc-treated mice using a negative-selection mouse NK cell enrichment kit (StemCell Technologies) and determined NK cell activity by a calcein-release assay using calcein-AM-labeled YAC-1 cells, as previously described^34^ with some modifications. The Calcein release assay revealed that, both in uninfected state and at 1 dpi, NK cell activity of the GlcNAc group was significantly higher than that of PBS group in blood (*P* = .0001, *P* = .0124) (Figure 6a) and spleen (*P* = .0428, *P* = .0010) (Figure 6c). In addition, although NK cell activity between the two groups has no significant difference in lung in the uninfected state, it was significantly higher in GlcNAc group than that in PBS group at 1 and 3 dpi (*P* = .0119, *P* = .0153) (Figure 6b). These findings suggest that GlcNAc not only induces NK cell proliferation, but also enhances NK cell activity.

The PBS and GlcNAc groups (*n* = 20 per group) were orally administered PBS or 1000 mg/kg GlcNAc for 1 week and infected with H7N9 influenza virus GX. At 0, 1, 3, and 5 dpi, five mice were randomly selected from each group and euthanized. NK cells were isolated from peripheral blood(a), lung(b), and spleen(c) and their activities were measured by a calcein-release-assay. (d) Experimental setup for NK-cell adoptive transfer. SPF mice were divided into PBS and GlcNAc groups and treated as described above. NK cells were purified from the lungs of mice in the two groups and then 200 µL PBS containing 1 × 10^6^ NK cells were injected i.v. into recipient mice (*n* = 10 per group) via the tail vein. The recipient mice in the control group were injected with an equivalent volume of PBS. All recipient mice were intranasally inoculated with 1 × 10^4^ EID_50_ H7N9 influenza virus. (e) Survival rate and (f) body weight of infected mice. SPF mice were divided into PBS and GlcNAc groups and treated as described above. (g) After treatment with PBS or anti-asialo GM1 antiserum, NK cells in peripheral blood and the lung were examined by flow cytometry after one day. On day 0, mice were infected with 1 × 10^4^ EID_50_ GX influenza virus. (h) Survival and (i) body weight of mice per group were monitored daily for 14 d. Survival was assessed with a log-rank (Mantel-Cox) test. NK cell activity assay and body weight was assessed using a two-way ANOVA. Error bars show the mean ± SD for each treatment. All experiments were performed at least twice under similar conditions.

To evaluate the effects in vivo, we transferred NK cells isolated from mice treated with PBS or GlcNAc to naive or infected mice (Figure 6d). NK cells were purified from the lungs by negative selection prior to transfer and flow cytometry confirmed that the purity of the transferred NK cells was greater than 80% (Fig. S10). Compared with the PBS-NK cell transfer group, GlcNAc-NK cell transfer improved the survival rate of infected mice by 20% (Figure 6e) and significant reduced weight loss at 9 dpi (*P* = .047) (Figure 6f). Furthermore, anti-asialo GM1 was employed to deplete NK cells before infection. Depletion of CD3^−^CD49b^+^ cells in the blood and lungs was confirmed by flow cytometry (Figure 6g). In addition, we found that anti-asialo GM1 had no significant effect on CD4+ and CD8+ T cells in vivo (Fig. S11). Although there was no difference in body weight between the groups (Figure 6i), compared with the PBS+PBS group, a 40% increase in survival rates was observed in mice that received an oral administration of GlcNAc and intraperitoneal injection of PBS (30% vs 70%) (Figure 6h). However, the survival curve indicated that the beneficial effects disappeared when NK cells were depleted (10% vs 10%) (Figure 6h). Based on these results, GlcNAc exerts anti-influenza effects mainly by regulating NK cells.

### GlcNac was generated by several gut microbes during influenza infection

**Figure 7.**
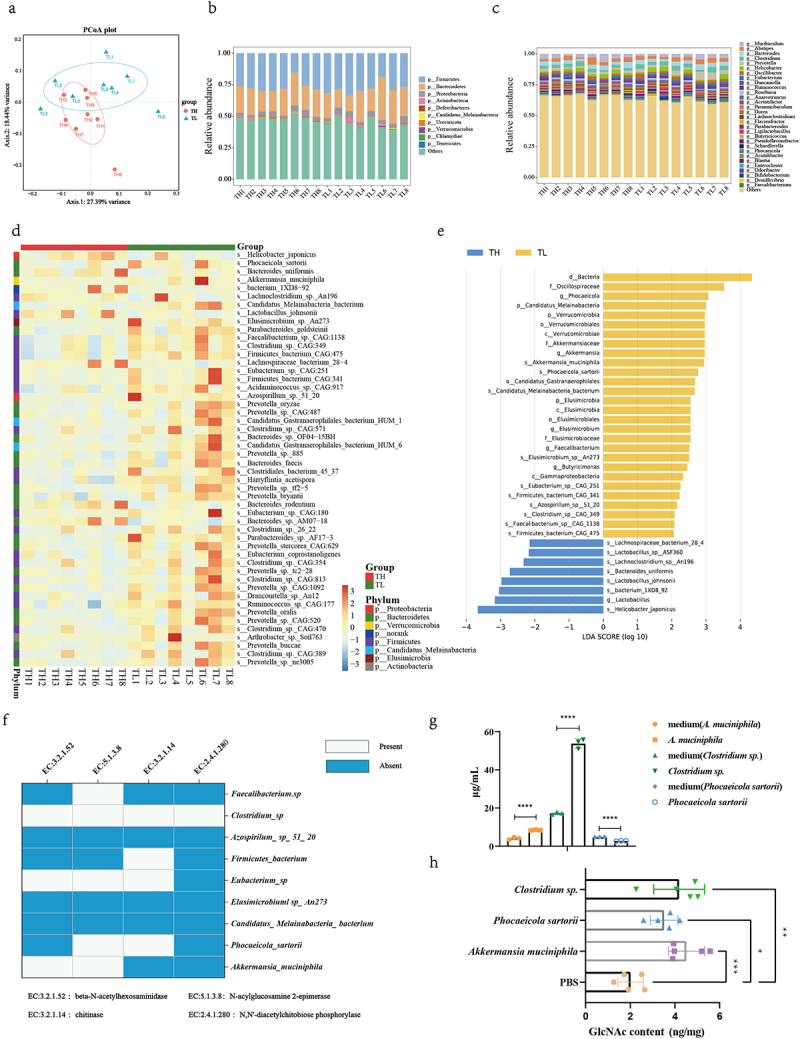
Upregulation of specific microbiota increased GlcNAc production in the gut.

GlcNAc is the basic unit of various polysaccharides in various organisms, including bacteria and fungi. Some bacteria can produce GlcNAc by degrading chitin^35^ or oligosaccharide chains of gastrointestinal mucins.^36–38^ We hypothesized that specific bacteria produce GlcNAc during infection. We conducted metagenomic sequencing of fecal microbiota in the TL and TH groups and assessed community structure and microbe abundance at different taxonomic levels using a PCoA. Although there was no difference in community structure (at phylum level) between the infected groups (Fig. S12a), there was a notable shift at the genus (Fig. S12b) and species levels (Figure 7a, Table. S8). The three phyla with the highest relative abundances in both groups were *Firmicutes*, *Bacteroidetes*, and *Proteobacteria* (Figure 7b, Table.S9). At the genus level, *Muribaculum*, *Alistipes*, *Bacteroides*, *Clostridium*, and *Prevotella* were dominant (Figure 7c, Table. S10).

Cecal content was sampled for the TH and TL groups for metagenomic analysis. (a) PCoA of the weighted UniFrac distances between the TH and TL group at the species level. The relative abundance of the (b) top 10 phyla and (c) top 30 genera in the TH and TL groups. (d) Hierarchically clustered heatmap analysis at the genus level; the relative values for bacterial genera correspond with color intensity. (e) The taxa with different abundances between the TH (blue bars) and TL group (yellow bars). The length of the histogram represents the influence size of the different species (LDA score > 2.0). (f)KEGG pathway enrichment analysis revealed the enzymes regulating GlcNAc production in differentially expressed microbiota. Quantitative determination of GlcNAc in cecal contents by UHPLC – MRM-MS in vitro (g) and in vivo (h). Data were assessed using a Wilcoxon – Mann–Whitney test. Statistics for GlcNAc determination in vitro using t-test and in vivo was one-way ANOVA. *P < .05, **P < .01, ***P < .001, ****P < .001. Error bars show the mean ± SD for each treatment

To elucidate the candidate anti-influenza bacteria, we analyzed the top 50 species with statistically significant differences in relative abundance between the groups (*P* < .05). We identified 42 bacteria in the TL group that exhibited greater abundance than that in the TH group (*P* < .05), including *P. sartorii*, *A. muciniphila*, *Candidatus Melainabacteria bacterium*, *Elusimicrobium sp*. An273, and *Parabacteroides goldsteinii* (Figure 7d, Table. S11). In contrast, *Helicobacter japonicus*, *Bacteroides uniformis*, *Bacterium* 1XD8–92, and *Lactobacillus johnsonii* were more abundant in the TH group (*P* < .05). According to the LEfSe, the abundance of nine bacteria (LDA >2) were markedly higher in the TL than the TH group, including *A. muciniphila*, *P. sartorii*, and *C. M. bacterium* (Figure 7e, Table. S12).

We assessed the relationship between the relative abundance of these nine bacteria in the gut contents and absolute levels of GlcNAc, and found a strong positive correlation between the relative abundance of *A. muciniphila*, *P. sartorii*, *Firmicutes bacterium*, and *C. M. bacterium* and GlcNAc levels (Fig. S13) (*P* < .05) (Table. S13 and Table. S14). Based on a KEGG analysis, chitinase, β-N-acetyl hexosaminidase, N-acyl glucosamine 2-epimerase, and N,N’-diacetylchitobiose phosphorylase are key enzymes in the catabolism of chitin – an important pathway for GlcNAc production. We aligned the key enzymes to the genomic positions of the nine bacteria and found that *Faecalibacterium sp*., *Clostridium sp*., *F. bacterium*, *Eubacterium sp*., *P. sartorii*, and *A. muciniphila* were enriched in at least one of the key genes (Figure 7f). This suggests that these bacteria may contribute to the upregulation of GlcNAc and host resistance to influenza. Next, we quantified the GlcNAc content in the culture supernatant of *Clostridium sp., A*. *muciniphila*, and *P. sartorii* and observed a significant increase in GlcNAc content in the culture supernatant of *Clostridium sp*. and *A. muciniphila* after 48 hours of culture (*P* < 0.0001) (Figure 7g and Table. S16). On the contrary, the content of GlcNAc in the supernatant of *P. sartorii* decreased obviously. However, we administered mice with *Clostridium sp*., *P. sartorii*, and *A. muciniphila* by gavage respectively and found that the levels of intestinal GlcNAc were all increased significantly (*P* = .0018, *P* < .001, *P =* .026) (Figure 7h and Table. S16). Then, we further investigated the impact of these bacteria on NK cells. The results indicated a significant increase of NK cells in blood, lung, and spleen after gavage with the mixed three bacteria (*P* < .001, *P* = .006, *P =* .0012) (Fig. S14a). Although CD107a + NK cells had no significant difference between the two groups, IFN-γ + NK cells of the mixed bacteria group were significantly higher than that of PBS group in both blood and spleen (*P* = .048, *P =* .003) (Fig. S14b). Meanwhile, a similar result with IFN-γ + NK cells was also obtained in the NK cell activity detection experiment, i.e., NK cell activity was significantly increased in the mixed bacteria group compared with that in PBS group in both blood and spleen (*P* < .001, *P* = .0011) (Fig. S14c). Our findings indicate that specific bacteria, including *Clostridium sp*., *P. sartorii*, and *A. muciniphila*, can directly or indirectly augment the expression of intestinal GlcNAc, thereby enhancing host influenza resistance by modulating NK cell.

## Discussion

Gut microbiota play a vital role in determining the outcomes of influenza virus infections.^24,39^ Although the mechanism by which microbiota exert their beneficial effects remains unclear, evidence indicates that specific microbial metabolites can mediate resistance against viral infection.^40,41^ Therefore, it is valuable to establish an effective screening strategy to identify new microbial metabolites with anti-influenza effects. Because of the complex and diverse composition of microbial metabolites, it remains challenging to exclude invalid interference factors and target identification of functional metabolites. The host response to infection typically exhibits considerable heterogeneity, the main reason being that individuals vary in their capacity for viral resistance and disease tolerance.^42^ Accumulating evidence suggests that this heterogeneity can be attributed to host gut microbiota and metabolites,^43,44^ pointing out the possibility of reverse screening specific functional metabolites by combining host infection heterogeneity with metabolomic analysis of intestinal contents. In the present study, we established an effective strategy to identify anti-influenza gut metabolites by considering host resistance (TH group, low resistance; TL group, high resistance) with metabolomic analysis of intestinal contents. We successfully identified several potential anti-influenza gut metabolites and demonstrated that GlcNAc and adenosine exerted anti-influenza effects in vivo and in vitro, respectively. Although some technical problems inherent in metabolomic analysis may lead to the omission of important information,^45^ our data are sufficient to prove that our approach is a scientific and effective strategy for screening specific anti-influenza metabolites from gut metabolites. Notably, viral resistance is not a unique factor that can be considered, which also supports the previous viewpoint that any other factor that introduces heterogeneity in the host response to infection can be used.^39^ Therefore, disease tolerance may be a good entry point. Our research confirms that it is feasible to understand the interaction between pathogens and hosts based on inherent heterogeneity perspective. Future research should employ this approach with disease models other than influenza infections in mice.

A growing number of studies have demonstrated the important roles of gut microbial metabolites in the pathogenesis of various diseases,^46^ suggesting that they could be important entry points to reveal the mechanisms by which gut microbes interact with the host. Specific intestinal metabolites, such as short-chain fatty acids,^41^ deaminotyrosine,^40^ and CoA,^39^ exert anti-influenza effects through different mechanisms. However, most studies have screened metabolites with anti-influenza potential by apply exogenous administration of known metabolites, which is unable to understand the relationship between changes in gut metabolites during infection and the host immune response to infection. We found that changes in specific gut metabolites were closely related to the viral clearance capacity in the host lung. Moreover, we successfully identified two novel anti-influenza gut metabolites, GlcNAc and adenosine, and found that the host can enhance viral resistance by upregulating endogenous GlcNAc in the gut during infection. Taken together, our data not only enrich our knowledge about the anti-influenza effect of gut metabolites, which is crucial to deepening our understanding of the lung-gut axis, but also provides new insights into the generation of heterogeneity in the host infection response.

NK cells are important immune cells and are traditionally regarded as the first line of defense against certain viral infections and tumors.^47^ However, it remains unclear whether microbiota can regulate their function. Several studies have shown that GlcNAc can exert anticancer effects by promoting the proliferation and activation of NK and T cells^48,49^ the role of GlcNAc in pathogen-induced infection has not been reported. Our study showed that oral administration of GlcNAc increased the proportion and killing activity of NK cells before and after influenza infection, thereby protecting the host from infection. Although the role of NK cells in influenza infection is complex and varies with viral dose,^50^ a study in H_7_N_9_‑infected patients showed that higher levels of NK cells promote recovery during disease progression.^51^ Our study demonstrated that GlcNAc can significantly enhance the proportion and activity of NK cells in mice both before and after infection. Moreover, we observed that the anti-influenza effect of GlcNAc was no longer present when NK cells were depleted using anti-asialo GM1. A previous study demonstrated that anti-asialo GM1 can also deplete basophils while targeting NK cell,^52^ in fact, our study also found that anti-asialo GM1 depleted basophils (Fig. S15a), suggesting a potential influence of basophils. However, our results indicated that GlcNAc treatment can only affect NK cells but not basophils (Fig. S15b). These findings support that GlcNAc primarily exerts its anti-influenza effect through NK cells. Given that GlcNAc is a natural small nontoxic molecule with virtually no side effects,^53,54^ future studies should consider its potential as part of novel influenza treatments.

GlcNAc has great potential in the treatment of gastritis and inflammatory bowel disease,^55,56^ damaged cartilage,^35^ rheumatoid arthritis, and cancer.^49^ However, these conclusions were all based on the exogenous administration of GlcNAc and were unrelated to the gut microbiota. Here, we showed that endogenous GlcNAc was upregulated in the gut of infected individuals with high virus resistance, and we hypothesized that gut microbes contributed to these changes. Chitin is the second largest carbohydrate in nature after cellulose^57,58^ and can be degraded by bacteria to produce GlcNAc.^35^ Based on the four key enzymes in the chitin catabolism pathway, we deduce that *Clostridium sp*., *P. sartorii*, and *A. muciniphila* are significantly associated with the upregulation of GlcNAc in the gut. Next, *in vitro*, we confirmed that *Clostridium sp*. and *A. muciniphila* can directly produce GlcNAc. However, *P. sartorii* was unable to directly produce GlcNAc. Nonetheless, *in vivo* experiments demonstrated that these three bacteria were all able to increase GlcNAc levels in the intestines. This suggested that *P. sartorii* may interact with other bacteria to indirectly produce GlcNAc. Moreover, our findings revealed that *Clostridium sp*., *P. sartorii*, and *A. muciniphila* can enhance the ratio and activity of NK cells in mice after oral administration, similar to the effects of GlcNAc. Overall, these results indicated that *Clostridium sp*., *P. sartorii*, and *A. muciniphila* can enhance host influenza resistance by upregulating GlcNAc. However, further investigation is needed to determine whether the gut microbiota modulates antiviral NK cell responses through other immune cells or gut microbial metabolites.

We also demonstrated that adenosine inhibited the proliferation of multiple influenza virus subtypes in vitro. Although oral administration of adenosine did not provide protection against influenza infection, intraperitoneal injection of adenosine improved the survival of infected mice (Fig. S16). This difference may be attributed to the degradation of adenosine in the digestive tract compared with the circulatory system. The main clinical use of adenosine is the treatment of cardiovascular diseases.^59^ An increasing number of studies have found that adenosine analogs have great anticancer^60^ and antiviral.^61,62^ However, the anti-influenza mechanism of adenosine requires further exploration.

Our study provides strong empirical support for the anti-influenza effects of GlcNAc through the regulation of NK cell activity. We confirmed that the endogenous expression of GlcNAc increased after influenza infection, which was mainly regulated by gut microbiota, including *Clostridium sp*., *P. sartorii*, and *A. muciniphila*. Our findings suggest that gut microbes can enhance host resistance to influenza through metabolite production. Future studies should consider the effects of manipulating gut microbial community structure or the endogenous production of metabolites on host resistance and tolerance to influenza infection.

## Materials and methods

### Bacteria and virus

*Akkermansia muciniphila* ATCC BAA-835 and *Clostridium sp*. BNCC195293 were obtained from BeNa Culture Collection (Suzhou, China). *Phocaeicola sartorii* JCM 17,136 was obtained from Biobw Culture Collection (Beijing, China). *A. muciniphila* was streaked on BD Brain Heart Infusion (BHI) agar (Becton Dickinson, Heidelberg, Germany) supplemented with 0.25% mucin. *Clostridium sp*. were cultured in Columbia (Qingdao Hope Biotechnology, Qingdao, China) supplemented with 5% sterile defidrinated Sheep Blood. *P. sartorii* was cultured in TSA (Becton Dickinson) supplemented with 5% sterile defidrinated Sheep Blood for 48 h under anaerobic conditions at 37°C.

Influenza viruses A/chick/Guangxi/YL01/2017 (H_7_N_9_), A/Puerto Rico/8/1934 (H_1_N_1_, PR8), and green fluorescent protein (GFP) recombinant H_5_N_6_ (A/duck/Hubei/WH18/2015) were propagated in the allantoic sac of 10-day-old chicken embryos and purified as previously described^63^. Purified influenza viruses were kept frozen at − 80°C until use. The HA cleavage motifs of H7N9 and H5N6 viruses met the criteria for a highly pathogenic avian influenza virus, showing high pathogenicity in mice.

### Animals

Specific pathogen-free (SPF) female C57BL/6J mice (8-week-old) were obtained from the Experimental Animal Center of China Three Gorges University. All animals were maintained in the same room and generally in the same facilities, under specific pathogen-free (SPF) conditions and had access to food and water ad libitum. All animal experiments were performed under animal biosafety level 3 (ABSL3) conditions. All animal experimental protocols were performed in accordance with the Guide for the Care and Use of Laboratory Animals Monitoring Committee of Hubei Province, China, and the protocol was approved by the Scientific Ethics Committee of Huazhong Agricultural University (Permit Number: HZAUMO-2019-018).

### Antibiotic treatment

SPF mice received an antibiotic solution (ATB), as previously described,^64^ with minor modifications. Ampicillin (1 mg/mL), streptomycin (5 mg/mL), vancomycin (0.25 mg/mL), and colistin (1 mg/mL) (Sigma-Aldrich, St. Louis, MO, USA) were added to the sterile drinking water of mice for 3 d and was stopped on the day preceding intestinal metabolite transplantation (IMT).

### Mouse cecum metabolite extraction and transplantation

A total of 40 C57BL/6 mice were divided into a normal/control (NC) (simulated phosphate buffered saline infection, *n* = 10) and H_7_N_9_ infection group (*n* = 30). All mice were anesthetized by intraperitoneal injection with a mix of ketamine/xylazine (100 and 5 mg/kg, respectively) in 100 μL of sterile phosphate-buffered saline (PBS).^65^ The infection group was intranasally inoculated with 1 × 10^4^ EID_50_ of H_7_N_9_, while the NC group was inoculated with an equal volume of PBS. Mouse cecum contents were harvested 5 d after influenza infection and immediately stored at − 80°C. Intestinal metabolite extraction was performed as previously described.^31^ The lungs of infected mice were collected, placed in PBS, homogenized using ceramic beads, and sorted according to their viral content: 20 samples were equally divided into a high (samples in the upper third of viral load, TH group) and low virus titer group (samples in the bottom third of viral load, TL group). Similarly, the cecum contents were divided into TH and TL groups. For the IMT experiment, the intestinal contents of the NC, TH, and TL groups were separately suspended in PBS and homogenized. The samples were centrifuged for 10 min at 4,000 *g* and 4°C, 30 min at 14,000 *g* and 4°C, the supernatant was harvested, and passed through a 0.22 mm filter. The microbial metabolites were then added to 15 mL drinking water.

Antibiotic-pretreated mice (*n* = 48) were randomly assigned to four groups (*n* = 12 per group): Mock, NC donor, TH donor, and TL donor. Each mouse received a daily oral administration of 200 μL NC, TH, or TL microbial metabolites, or PBS. After 5 d, all mice were anesthetized by intraperitoneal injection with a mix of ketamine/xylazine (100 and 5 mg/kg, respectively), the mice of NC donor, the mice of TH donor and TL donor were intranasally inoculated with 50 μL of PBS containing 1 × 10^4^ EID_50_ of H_7_N_9_ virus and the Mock group mice were inoculated with PBS. After 7 d, two randomly selected mice from each group were euthanized (The mice were sacrificed under ketamine-xylazine anesthesia) for lung sample collection and histological examination. We monitored the survival and body weights of the remaining mice in each group for 15 d (0–14 dpi).

### Mouse infection for fecal analysis experiments and sample collection

SPF mice (*n* = 38) were divided into an NC group (simulated PBS infection, *n* = 8) and H_7_N_9_ infection group (*n* = 30). All mice were anesthetized by intraperitoneal injection with a mix of ketamine/xylazine, the infection group were intranasally inoculated with H_7_N_9_ (1 × 10^4^ EID_50_) and the NC group mice were inoculated with PBS. After 5 d, cecum content samples were collected and stored at − 80°C; lung tissue was harvested, placed in PBS, homogenized, and sorted according to viral content (*n* = 8 for the TH and TL group, respectively). Cecum contents of the NC, TH, and TL groups were used for metabolomic and metagenomic analyses.

### In vivo treatment of mice with specific metabolic compounds

To evaluate the anti-influenza effect of eight specific metabolic compounds, antibiotic-pretreated mice received a daily oral administration (200 μL) of GlcNAc (1000 mg/kg), adenosine (2 mg/kg), L-arginine (500 mg/kg), cellobiose (20 mg/kg), maltose (3000 mg/kg), citraconic acid (200 mg/kg), malonic acid (400 μg/kg) or Glu-Pro (20 mg/kg), or PBS for 1 week. Mice were anesthetized as above and then intranasally inoculated with H_7_N_9_ (1 × 10^4^ EID_50_, *n* = 10 mice per group). The survival and body weights of the remaining mice per group were monitored daily for 15 d.

To assess disease progression at 0, 3, and 5 dpi, 48 antibiotic-pretreated mice were randomly assigned to two groups (*n* = 24): a PBS and GlcNAc groups. Mice in the PBS group were orally administered 200 μL of PBS, and those in the GlcNAc group were administered 1000 mg/kg GlcNAc per day. Influenza infection was performed as described above. After 0, 3, and 5 d, the lungs of five mice from each group were collected to determine virus titers and cytokine levels. For the histological examination, the remaining mice per group were euthanized at 0, 3, and 5 d after infection (*n* = 3 at each time point); their lungs were collected and fixed with 4% polymethylene formaldehyde.

To investigate the function of NK cells in the anti-influenza effect of GlcNAc, 50 SPF mice were divided into three groups: Mock (*n* = 10), PBS (*n* = 20), and GlcNAc group (*n* = 20). Gavage was performed as described above and 10 mice were randomly selected from PBS and GlcNAc group for NK cell depletion. After 1 d, each mouse was intranasally infected (1 × 10^4^ EID_50_) and the Mock group mice were inoculated with PBS. The body weight and survival of each group were monitored daily for 14 d.

### Gut colonization with commensal species and sample collection

The gut of antibiotic-pretreated SPF mice was treated with specific bacterial strains. *A. muciniphila*, *Clostridium sp*., and *P. sartorii* were separately cultured in anaerobic conditions at 37°C for 48 h, harvested by centrifugation (5,000 rpm, 5 min, 4°C), and resuspended in PBS at a concentration of 1 × 10^9^ CFU/mL. The antibiotic-pretreated mice (*n* = 20) were randomly divided into four groups (*n* = 5 per group) and administered the following by daily oral gavage: vehicle (sterile PBS, 200 μL/mouse, group 1), a suspension of *A. muciniphila* (200 μL/mouse, group 2), *Clostridium sp*. (200 μL/mouse, group 3), or *P. sartorii* (200 μL/mouse, group 4). After 1 week, mouse cecum contents were harvested for ultra-high-performance liquid chromatography – multiple reaction monitoring-mass spectrometry (UHPLC – MRM-MS) analysis.

### Histopathology and immunohistochemistry

The harvested lungs were fixed with 4% paraformaldehyde and then paraffin embedded. Each paraffin-embedded tissue sample was sectioned into 5-µm-thick sections. Tissue section was deparaffinized, rehydrated, and stained with hematoxylin-eosin (HE) for the examination for histopathological analysis. The lung sections were assessed using an AxioVert 200 M (Zeiss, Oberkochen, Germany) optical microscope. Stained slides were scanned using a Pannoramic slide scanner (Pannoramic 250/MIDI) and the morphological changes were assessed by a semi-quantitative score. For the scoring, a dual histopathology scoring system adapted from^66,67^ was used to assess pathological severity. For each criteria, a score 0 = absent, 1 = 1–10% of lung section, 2 = 11–25% of lung section, 3 = 26–50% of lung section, and 4 = >50% of lung section affected.

Immunohistochemistry experiments were performed on the second section. Lung sections were washed in PBS (3 × 5 min), incubated in PBST (0.3% Triton X-100 in PBS) for 30 min at room temperature, and then washed with PBS (3 × 5 min). Sections were blocked in 5% normal goat serum in PBS-Tween (0.05%) for 1 h at room temperature. Primary antibodies (rabbit anti-influenza nucleoprotein [NP], dilution 1:500, GeneTex Irvine, CA, USA) were added and the samples were incubated overnight at 4°C. After washing with PBS (5 × 15 min), the sections were incubated with CoraLite594–conjugated Goat Anti-Rabbit IgG (dilution 1:400, Proteintech, Wuhan, China) at room temperature for 1 h. The sections were washed with PBS and mounted onto slides. Images were obtained a Pannoramic slide scanner (Pannoramic 250/MIDI).

### Metabolomic analysis of cecum contents

A global metabolomics analysis was conducted using gas chromatography-mass spectrometry (GC – MS) and ultra-performance liquid chromatography – MS (UPLC – MS). The GC – MS analysis was performed using an Agilent 7890B gas chromatograph equipped with PEGASUS HT time-of-flight mass spectrometer (LECO). UPLC – MS analysis was conducted using an Agilent 1290 Infinity UPLC and AB triple TOF 5600\/6600 mass spectrometer (AB SCIEX). Sample equivalents were mixed to prepare quality controls (QCs) for the assessment of reproducibility. Detailed sample preparations, GC – MS and UPLC – MS analyses, and QC preparation are described in the Methods section of Supplementary material. Ion peaks generated from GC – MS were annotated with LECO-Fiehn Rtx5 database, whereas those from UPLC – MS were annotated with the MetDDA database and LipDDA methods. All data were log-transformed for normalization before statistical analysis.

### Cell viability assay

A CCK-8 kit (Donjindo, Kumanoto, Japan) was used to measure the viability of drug-treated Caco-2 cells according to the manufacturer’s protocol. Caco-2 cells were seeded onto 96-well plates and cultured until 80% confluence was reached. The cells were treated with compounds at different concentrations for 24 h, after which 10 µL of CCK-8 solution was added and incubated at 37°C for 3 h. The absorbance at 450 nm was measured using a Spark 10 M (Tecan Austria GmbH Untersbergstr, Grödig, Austria).

### Cell culture and treatments

Caco-2 cells were cultured in DMEM (Gibco 11,320–033) containing 10% fetal bovine serum (FBS) (PNA-Biotech, Germany) in 5% CO_2_ at 37°C. Caco-2 cells were seeded on 12-well plates at 1 × 10^5^ cells/well until a confluence of 80% was reached. Different final concentrations were added to the culture medium for 12 h. The treated cells were infected with influenza virus at an MOI of 0.1 for 1 h, followed by two washes with PBS. The corresponding concentration of the compound was added and incubated with the infected cells. The cell culture supernatants were harvested at different time points (12, 24, and 36 h) post-infection to quantify virus RNA (vRNA) and determine the 50% tissue culture infective dose (TCID_50_). The cell pellets were subsequently subjected to western blotting.

### Western blotting

Cells were lysed on ice in mammalian protein extraction reagent. Western blotting was conducted as previously described.^68^ Cell lysates were separated by SDS-PAGE and transferred to nitrocellulose. The antibodies used in the western blot assays were as follows: The rabbit anti-influenza NP protein antibody (dilution 1:3000, GeneTex, Irvine, CA, USA) and the rabbit anti-GAPDH antibody (dilution 1:3000, Cali-Bio, Coachella, CA, USA). Bound Abs were detected using an HRP-conjugated anti-rabbit IgG secondary Ab (dilution 1:5000, ZSGB-BIO, Beijing, China) and ECL development (Pierce) according to the manufacturer’s protocol.

### TCID_50_ analysis

We performed titration by TCID_50_-hemagglutination (TCID_50_-HA) assay on MDCK cells, as previously described. MDCK cells were seeded on a 96-well plate, the supernatants of infected cells were serially diluted in DMEM (Sigma-Aldrich) and then adsorbed for 1 h onto MDCK cells in 96-well plates. The inoculum was removed and the cells were washed with PBS and maintained in fresh DMEM. The MDCK cells were incubated at 37°C for 72 h, and virus titers were determined by calculating log_10_ TCID_50_/mL using the Spearman – Karber method.

### Titration of the viral load in lung samples

To determine the virus titers, 10-d-old SPF embryonated chicken eggs were inoculated with diluted supernatants of lung homogenate (as described for mouse lung sample preparation). After 72 h at 37°C, the allantoic fluid was harvested for hemagglutination. Virus titers were calculated using the method described by Reed and Muench.^69^

### Determination of cytokine concentration

The concentrations of cytokines, including IFN-β, IFN-γ, IL-1β, TNF-α, IL-6, and IL-10, in the supernatants of the lung homogenates and serum were detected using an enzyme-linked immunosorbent assay (ELISA) kit (NeoBioscience Technology Company, Shenzhen, China).

### RNA extraction and qRT-PCR

To quantify the vRNA in infected lung tissue, whole lungs were homogenized in PBS (1 mL/lung), the homogenate was centrifuged at 8000 rpm for 5 min, and the supernatants were stored at −80°C until use. Total RNA was extracted from the supernatants using TRIzol (Invitrogen) according to the manufacturer’s instructions. vRNA was reverse-transcribed with AMV reverse transcriptase (Takara Bio, Japan) using the U12 primer. qRT-PCR was performed using an ABI ViiA 7 PCR system (Applied Biosystems, USA) and a SYBR Green PCR Kit (Roche, Switzerland). The pCAGGS-NP plasmid was used to generate a standard curve with which the number of vRNA were calculated. The relative NP mRNA level of IAV was normalized to GAPDH mRNA and analyzed by the threshold cycle calculation method (2^−ΔΔCT^). The primers used for qRT-PCR are listed in Table S1 and S2.

### Determining CD4+ T cell, CD8+ T cell, and NK cell responses in vivo

SPF mice (total number 40) were randomly assigned to two groups (*n* = 20): a PBS and GlcNAc group. Mice in the PBS group were orally administered 200 μL PBS, and those in the GlcNAc group were administered 1000 mg/kg GlcNAc per day. Influenza infection was performed as described above. At 0, 1, 3, and 5 dpi, the blood, spleen, and lungs of three mice from each group were collected to determine CD4+ T cell, CD8+ T cell, and NK cell responses by flow cytometry.

### Isolation of peripheral blood mononuclear cells, lung lymphocytes, and spleen mononuclear cells

Blood samples were anti-coagulated with Na_2_EDTA and resuspended in 0.84% NH_4_Cl solution to lyse the red blood cells (RBCs). Peripheral blood mononuclear cells (PBMCs) were collected after washing twice with PBS and the number of cells was calculated using cell count plates.

Lung lymphocytes were isolated as previously described^70^ Mice were exsanguinated from the orbital cavity to minimize the amount of blood in the lungs. To obtain a single-cell suspension, the lungs were removed and passed through a cell strainer (BD Falcon). The cells were resuspended in 35% Percoll solution (in PBS buffer) and centrifuged at 1,500 rpm for 15 min at room temperature. Red blood cells in the lymphocyte pellet were lysed with 0.8% NH_4_Cl and washed twice with PBS.

Spleens were removed and passed through a cell strainer (BD Falcon) to obtain a single cell suspension, and the spleen leukocytes were isolated with 0.84% NH_4_Cl to lyse erythrocytes.

### Flow cytometry

To determine NK cell responses in blood, spleen and lung single-cell suspensions were stained as previously described, with minor modifications.^71,72^ We incubated single-cell suspensions in 1.5 mL Eppendorf tubes for 2 h at 37°C in a medium containing brefeldin A. Cells were suspended in buffer and incubated with anti-CD16/CD32 mAb for 30 min at room temperature. The cells were then stained for surface markers, fixed, permeabilized, and intracellularly stained for cytokines, according to the manufacturer’s instructions. The antibodies and reagents used for flow cytometry were as follows: PE-anti-mouse CD49b (Cat. #108907), FITC-anti-mouse CD3 (Cat. #100204), PE-anti-mouse-CD8a (Cat. #100707), APC-anti-mouse-CD4 (Cat. #100412), anti-mouse CD16/32 (Cat. #101320), APC-anti-mouse-CD107a (Cat. #121613), APC anti-mouse FceRla (Cat. #134316) and PE/Cyanine7-anti-mouse IFN-γ (Cat. #505825, BioLegend). Stained cells were analyzed by flow cytometry using a Cytoflex-LX flow cytometer (Bekeman). Data were analyzed using FlowJo software (Tree Star).

### Purification of NK cells

NK cells were purified using an EasySep Mouse NK Cell Isolation Kit (StemCell Technologies) according to the manufacturer’s instructions. Single-cell suspensions of lungs or spleen (1 × 10^6^ cells/mL) were treated with RPMI supplemented with 2% FBS and penicillin-streptomycin. Cells (2 mL) were transferred to a 5-mL tube, mixed with 100 µL isolation cocktail, and incubated at room temperature for 10 min. Pre-vortexed RapidSpheres (200 µL) were added, mixed, and incubated for 5 min at room temperature. The tube was placed (without its lid) onto a magnet, incubated, and inverted, and the enriched NK cell suspension was transferred to a new tube. Finally, the total nucleated cell count was determined using a Neubauer hemocytometer.

### NK-cell adoptive transfer

Ten SPF mice were randomly assigned to two groups (*n* = 5): a PBS and GlcNAc group. Gavage was performed as previously described. Mouse primary NK cells were purified from C57BL/6 mouse spleens using the EasySep Mouse NK Cell Isolation Kit (StemCell) and 200 µL PBS containing 1 × 10^6^ NK cells was injected intravenously into recipient mice (*n* = 10 per group) via the tail vein.^50^ Recipient mice in the control group were injected with an equivalent amount of PBS. On the same day, all recipient mice were anesthetized by intraperitoneal injection with a mix of ketamine/xylazine and intranasally inoculated with 1 × 10^4^ EID_50_ H_7_N_9_. The survival rate and body weight of the infected mice were monitored daily.

### Depletion of NK cells

Forty-four mice were orally administered PBS or GLcNAc for one week, and then they were divided into four groups: PBS+PBS, PBS+anti-asialo GM1, GlcNAc+PBS, and GlcNAc+anti-asialo GM1 groups. Depletion of NK cells were performed as previously described.^70^ B6 mice were treated with 50 μL anti-asialo GM1 (Wako Chemicals) 1 d before infection. Other mice were injected with PBS by intraperitoneal injection. NK cell depletion was verified by flow cytometry to measure the percentage of NK cells in the lungs.

### NK cell activity assay

NK cell activity was determined by calcein release test using calcein-AM-labeled YAC-1 cells as target cells. To label target cells with calcein-AM, 1 × 10^6^ YAC-1 cells were resuspended in RPMI-1640 medium containing 5 µM calcein-AM and incubated at 37°C for 30 min. The marked target cells (1 × 10^4^ cells/well) were transferred to a 96-well plate. The separated NK cells were added to the plate at an effector-to-target (NK:YAC-1) ratio of 50:1 and incubated for 4 h at 37°C. To determine calcein release from calcein-AM-labeled YAC-1 cells, the fluorescence intensity of the harvested supernatant was measured at 485 nm excitation and 530 nm emission using an Infinite M200 PRO microplate reader (Tecan, Männedorf, Switzerland). Spontaneous release was determined by fluorescence in target cell areas where effector cells were absent; maximum fluorescence release was determined by treatment of labeled target cells with 1% Triton X-100. NK cell activity (killing%) was calculated as follows: Specific Release (%) = 100 × ([Experimental Fluorescence Release] – [Spontaneous Fluorescence Release])/([Maximum Fluorescence Release] – [Spontaneous Fluorescence]). All trials were performed in triplicate.

### Detection of GlcNAc in intestinal contents by UHPLC – MRM-MS

An aliquot of each individual sample was weighed and transferred to a 2 mL Eppendorf tube. After the addition of 1000 μL extraction solution (acetonitrile: methanol: water at 2:2:1), the samples were vortexed for 30 s, homogenized at 40 Hz for 4 min, and sonicated for 5 min in an ice-water bath. The homogenate and sonicate cycle were repeated three times, and centrifugation at 12 000 rpm and 4°C for 15 min. An aliquot of the clear supernatant was diluted another 10 times for UHPLC – MS/MS analysis.

UHPLC separation was carried out using an Agilent 1290 Infinity II series UHPLC System (Agilent Technologies) equipped with an Agilent ZORBAX Eclipse Plus C18 (2.1 mm × 150 mm, 1.8 μm). Mobile phase A was 0.2% formic acid in water and mobile phase B was methanol. The elution gradients are listed in Table. S3. The flow rate was set to 300 μL/min, column temperature was set to 35°C, the auto-sampler temperature was set to 10°C, and the injection volume was 1 μL. We used an Agilent 6495 triple quadrupole mass spectrometer (Agilent Technologies) equipped with an AJS electrospray ionization (AJS-ESI) interface for assay development. The ion source parameters were as follows: capillary voltage = −2500 V, nozzle voltage = −1500 V, gas (N_2_) temperature = 250°C, gas (N_2_) flow = 11 L/min, sheath gas (N_2_) temperature = 400°C, sheath gas (N_2_) flow = 12 L/min, and nebulizer = 35 psi. The MRM parameters for the targeted analytes were optimized by injecting a standard solution of individual analytes directly onto the API source of the mass spectrometer. At least two MRM transitions (i.e., the Q1/Q3 pairs) were obtained per analyte, and the two most sensitive transitions were used in the MRM scan mode to optimize the collision energy for each Q1/Q3 pair. Between the two MRM transitions per analyte, the Q1/Q3 pairs that showed the highest sensitivity and selectivity were used for quantitative monitoring (Table. S4). The additional transitions acted as qualifiers to verify the identity of the target analyte. Agilent MassHunter Work Station Software (B.10.00, Agilent Technologies) was used for MRM data acquisition and processing.

### DNA extraction and metagenomic library sequencing

Immediately after collection, all fecal samples were frozen at − 80°C and transported to the laboratory on ice. Bacterial DNA from fecal samples was extracted using an improved CTAB method.^73^ A Nanodrop 2000 spectrophotometer (NanoDrop Technologies, Wilmington, DE, USA), Qubit 3.0 fluorometer (Life Technologies, Carlsbad, CA, USA), and 0.8% agarose gel electrophoresis were used to detect DNA concentration and quality.

A qualified DNA sample (1 µg) was randomly broken into fragments of approximately 350 bp using a Covaris ultrasonic crusher, and a whole library was prepared by end repair, addition of a tail, addition of sequencing adapters, purification, and PCR amplification. We used Qubit 3.0 for preliminary quantification, diluted the library to 2 ng/µL, and then detected the insert size of the library with an Agilent 2100 (Agilent Technologies). qPCR was used to determine the effective concentration of the library. To ensure good quality of the library, the effective concentration of the library must be >3 nM.

### Determination of β-diversity indices

β-Diversity was calculated using weighted UniFrac distances and assessed using a principle coordinate analysis (PCoA). The Bray – Curtis distances between the samples were used for principal component analysis using the default function in R software (version 3.6.0) (The R Foundation, Vienna, Austria). For each grouping variable, a 95% confidence ellipse was calculated using the vegan and ape packages.

### Metagenomic assembly, gene catalogue generation, and gene abundance calculation

Qualified libraries were sequenced using an MGIseq2000. To generate clean data, we removed low-quality bases (quality value ≤ 38) exceeding a certain percentage of reads (default is 40 bp), N bases that reached a certain percentage of reads (default is 10 bp), and overlap with the Adapter exceeding a certain threshold (default is 15 bp). Host reads were filtered using Bowtie2.^74,75^ We linked the overlapped reads, without using the genome, with the splicing software idbaud – based on the de Bruijn graph (http://i.cs.hku.hk/~alse/hkubrg/projects/idba_ud/).^76,77^ We spliced and assembled valid sequences, iterating the k value from small to large to remove short and low-depth contigs based on the threshold. MetaGeneMark software^75^ was used for gene prediction with default parameters after removing sequences with gene lengths of less than 100 nt. The gene prediction results were mixed for each sample, and CD-HIT software^78,79^ was used to remove redundancies. The redundant gene catalog was clustered with 95% identity and 90% coverage parameters,^80^ and the longest gene in each class was taken as the representative sequence to construct a non-redundant gene set.

To determine gene abundance, the clean data for each sample was compared to the non-redundant gene catalog using Bowtie2 with the alignment parameters:^80,81^ –end-to-end, –sensitive, -I 200, -X 400. The number of reads was calculated for each gene per sample, and the genes that support ＜ = 1 number of reads were determined to obtain the absolute abundance information. Considering the influence of gene length and sequencing depth on abundance estimates, the absolute abundance information must be normalized as described above.^82,83^

### Taxonomic annotation and abundance profiling

The BLASTp command of Diamond software (v0.9.12.113)^84^ was used to align the protein sequences of the unigenes with the bacterial library of NR (non-redundant protein) (with E = 1^−5^), select the hit with the highest score as the final annotation result, the species annotation is obtained through the taxonomic information database corresponding to the NR library, and then the abundance of the species is calculated by using the sum of the gene abundances corresponding to the species. We calculated species abundance at the domain, kingdom, phylum, class, order, family, genus, and species level as the sum of the corresponding gene abundances.

### Statistical analysis

We used linear discriminant analysis effect size (LEfSe) to estimate the magnitude of the effect of abundance on differential effects at the species level (using a logarithmic linear discriminant analysis (LDA) score threshold of 2). We compared microbial community abundance at the different taxonomic levels using the wilcox.test (version 3.6.0).^85^

Survival rates were estimated by the Kaplan – Meier analysis, and homogeneity was evaluated using a log-rank test. Data from the mouse and cell models are expressed as mean ± SD. Statistical significance was tested by a two-tailed Student’s *t*-test, one-way ANOVA, or two-way ANOVA when appropriate. Statistical significance was set at *P* <.05. Analyses were performed using GraphPad Prism v9.0.0.121 (GraphPad Software, San Diego, CA, USA).

## Supplementary Material

Supplemental Material

## Data Availability

The datasets generated in the current study were deposited to the NCBISRA database under the BioProject accession PRJNA861413. The metabolomics datasets generated and analyzed during the current study are available in the MetaboLights repository, using accession number MTBLS5410, http://www.ebi.ac.uk/metabolights/MTBLS5410. Raw data for UHPLC – MRM-MS is publicly available upon acceptance at: https://doi.org/10.6084/m9.figshare.20377545.v1 and https://doi.org/10.6084/m9.figshare.24042927.v1. All other relevant data are available from the corresponding authors upon reasonable requests, after signing a data access agreement. Correspondence and requests for materials should be addressed to Meilin Jin or Qiang Zhang.
