# Supplementary material for: GlcNac produced by the gut microbiome enhances host influenza resistance by modulating NK cells
Source: Gut Microbes. 2023 Nov 12;15(2):2271620. doi: 10.1080/19490976.2023.2271620 (PMC10730189; doi:10.1080/19490976.2023.2271620)
Supplement: Supplemental Material [file KGMI_A_2271620_SM9125.zip › Supplementary Material.pdf]

Supplementary Figure 1

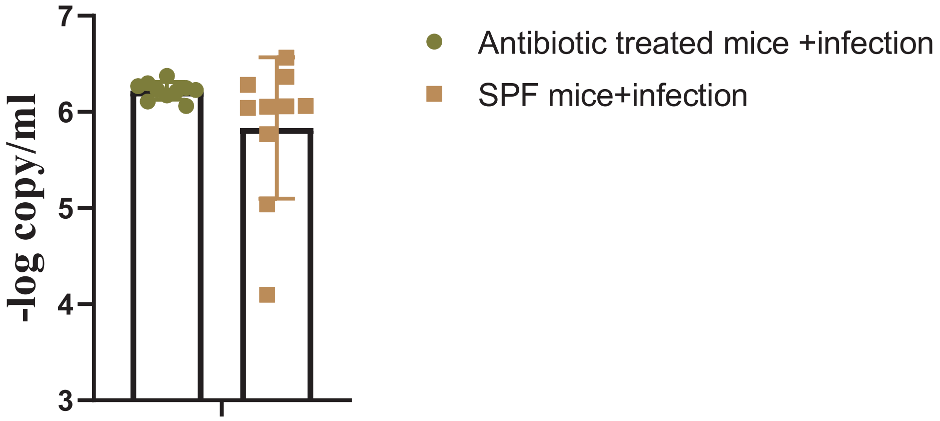

Fig. S1. The number of influenza virus vRNA copies in the lungs of mice was measured after treatment with or without antibiotics. Lungs of ATB or SPF mice were collected 5 d after GX infection (n = 10); The Copy Number of Influenza Virus vRNA were detectmined by qPCR.

Supplementary Figure 2

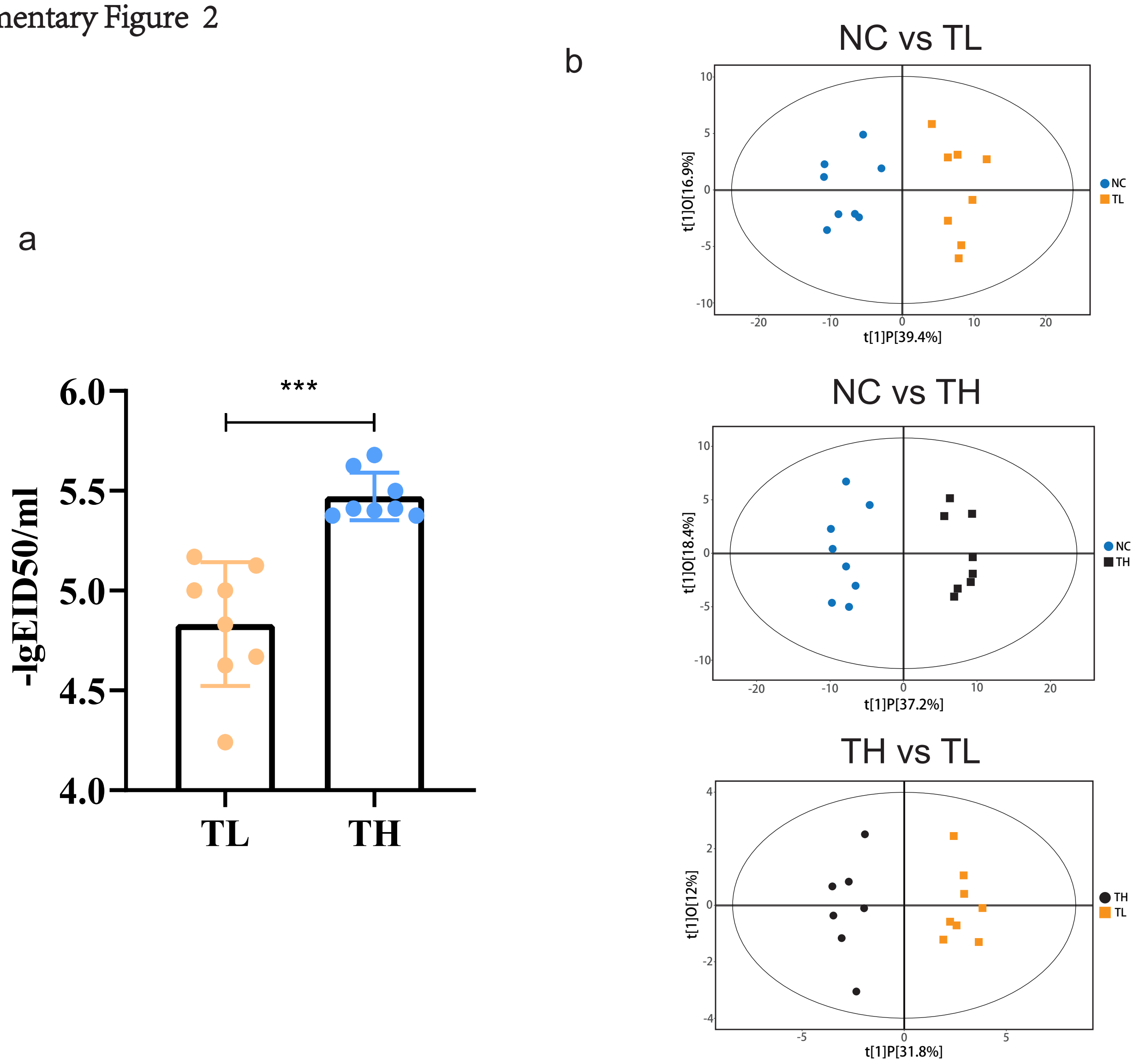

Fig. S2. (a)Viral loads in lungs of mice in TH and TL groups. Lungs of mice were collected 5 d after GX infection (n = 30); 16 of them were divided into high (n = 8, TH) and low virus titer (n = 8, TL) groups based on the level of viral load in the lungs. Viral loads determined by EID50. (b) OPLS-DA showing differences in metabolite composition between the TH, TL, and NC groups.Viral titer were assessed using Student's t -test ***P < 0.001

Supplementary Figure 3

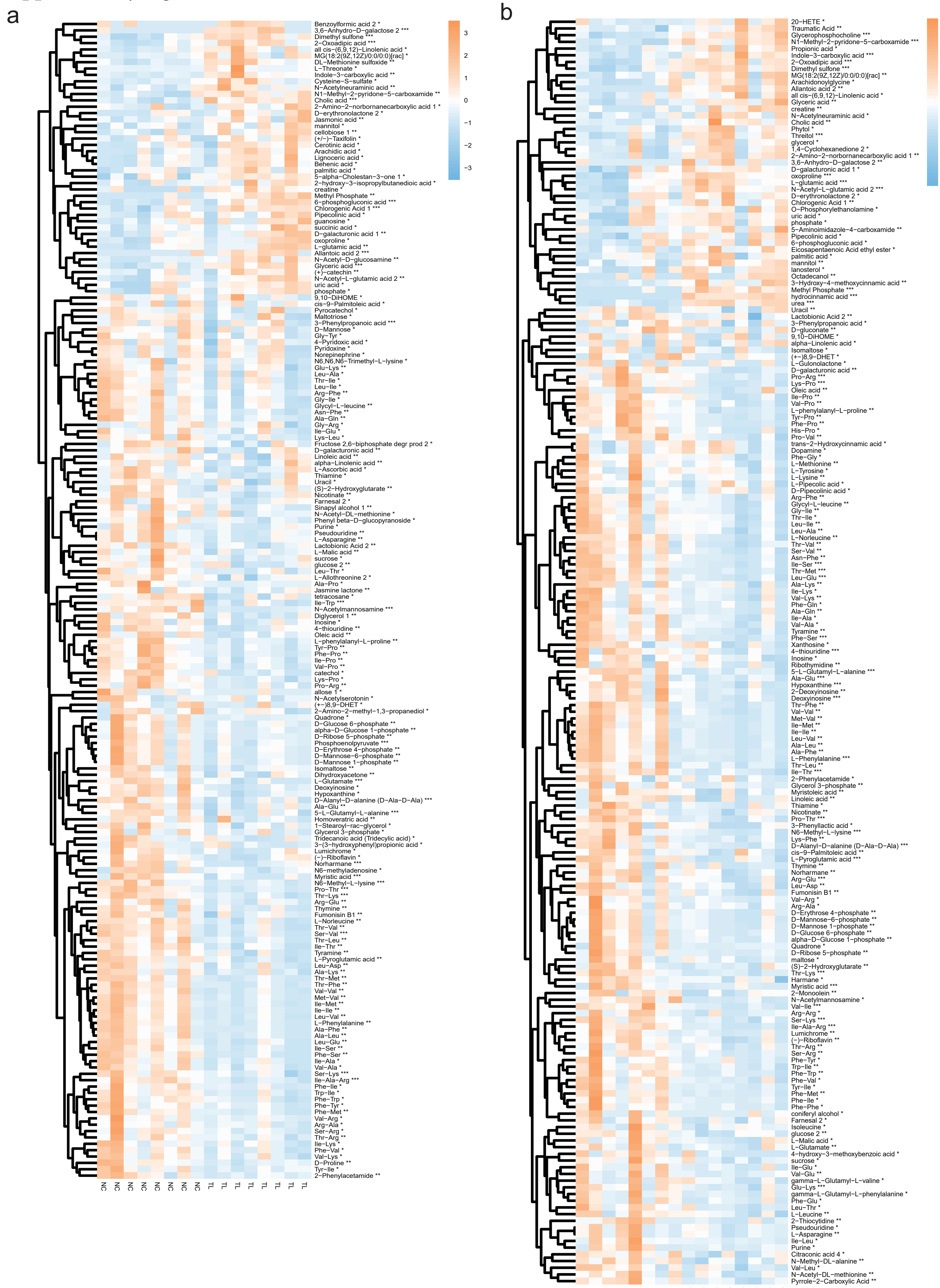

Fig. S3. Hierarchical clustering analysis of TL vs NC (a) and TH vs NC (b). Experimental description references to Fig. 2.

Supplementary Figure 4

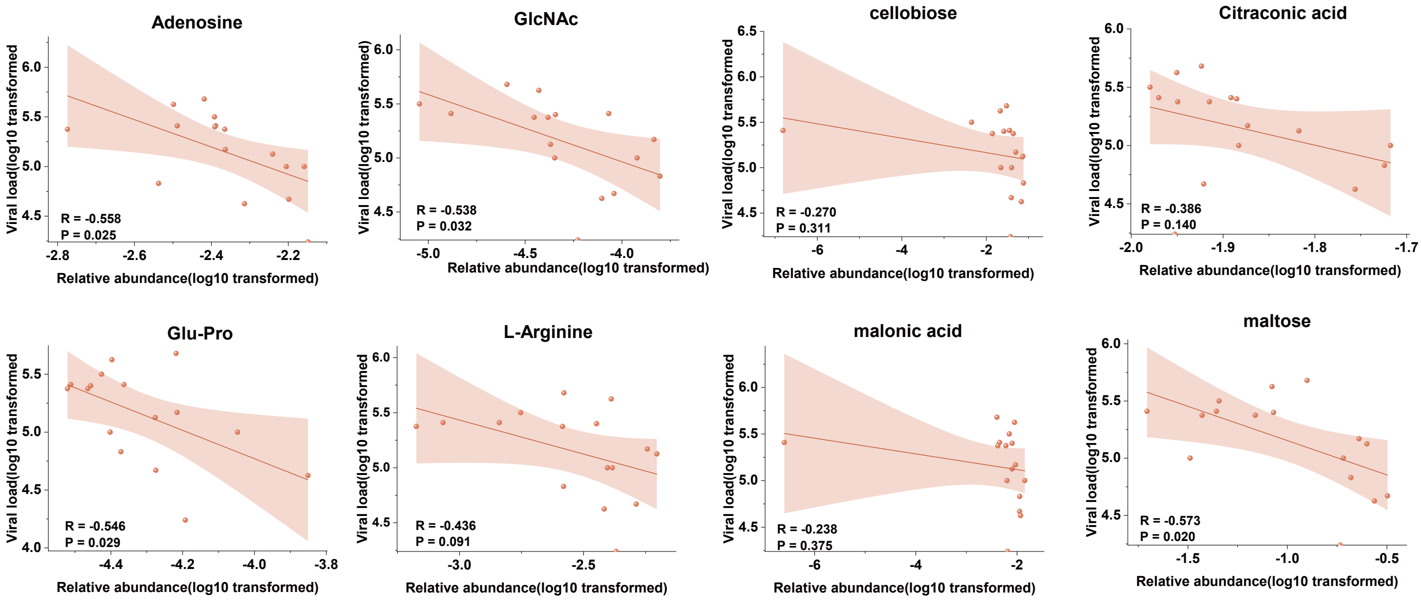

Fig. S4. Association of gut metabolites relative abundance and lung viral load. Correlation between gut metabolites relative abundance and lung viral load was examined using Spearman’s correlation test. Regression lines with 95% CI (grey area) were shown on scatter plots.

Supplementary Figure 5

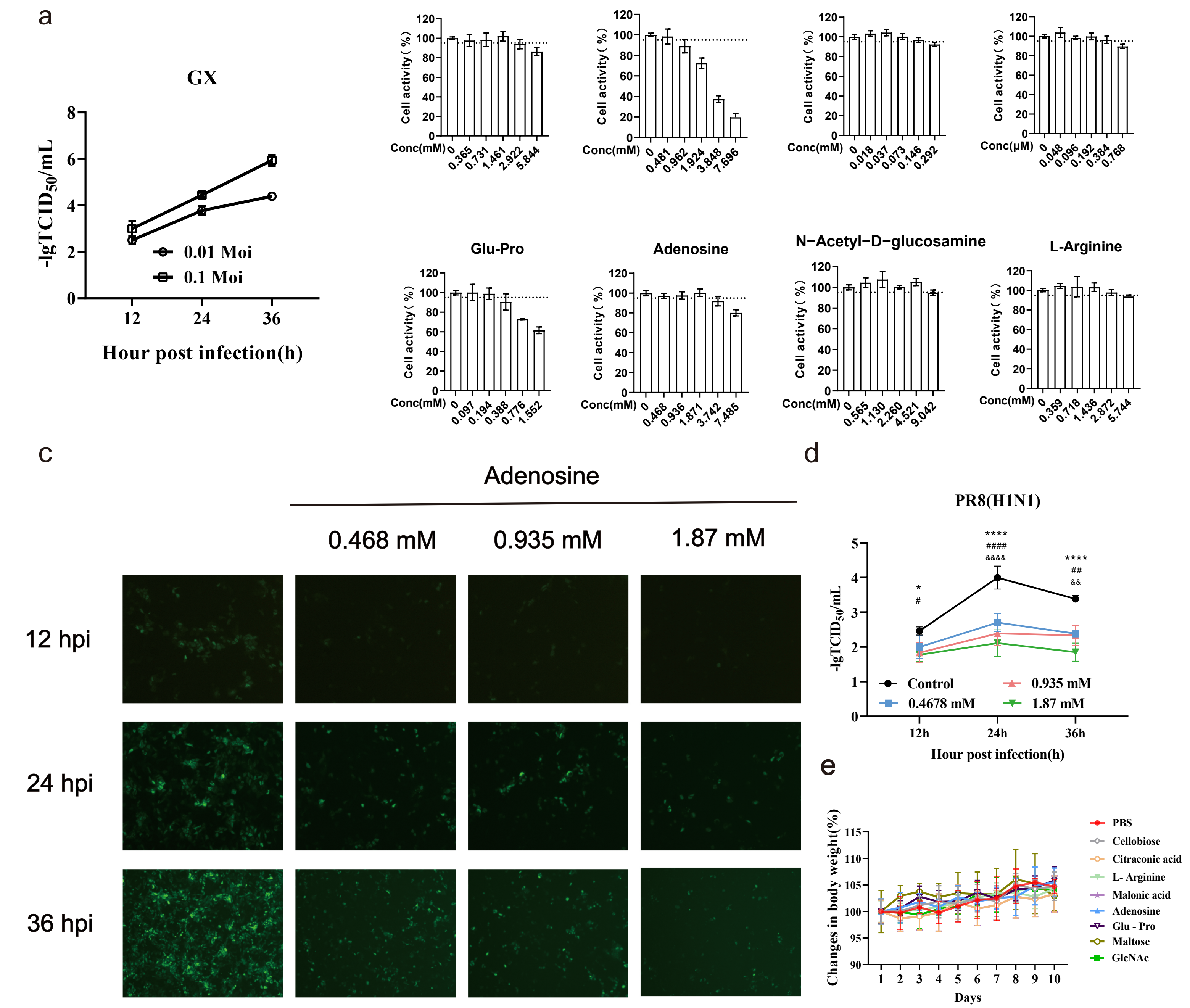

Fig S5. The anti-influenza effect of metabolites were evaluated In vitro and in vivo experiments. (A) Caco-2 cells infected with the GX at an MOI of 0.1. Cell supernatant samples were harvested 12, 24, and 36 h after infection.Virus tilter determined by TCID50. (B) Caco-2 cells were treated with 8 gut metabolites using the indicated concentrations for 24 h. Then, the cell viability was measured by CCK-8. (C) Caco-2 cells were plated in 12-well plates until the cell confluence reached 80%. Subsequently, at different final concentrations was added to the culture medium and cultured the cells for either 12 h. Then, the treated cells were infected with H5N6-GFP virus at an MOI of 0.1 for 1h, followed by treatment with adenosine at indicated concentrations for 12, 24 and 36 h. After that, the GFP intensity was acquired using fluorescence microscopy. (D) The treated cells were infected with H1N1 influenza virus at an MOI of 0.1 for 1h, followed by treatment with adenosine at indicated concentrations for 12, 24 and 36h, virus titer was determined by TCID50 assay. (E) SPF mice were treated with 8 metabolites (GlcNAc: 1000 mg/kg; adenosine: 2 mg/kg; L-arginine: 500 mg/kg; cellobiose: 20 mg/kg; maltose: 3000 mg/kg; citraconic acid: 200 mg/kg; malonic acid: 400μg/kg; Glu-Pro: 20mg/kg) or PBS via oral gavage per day. Body weights in each group were monitored daily for 10 days post-treatment. Statistical Analysisusing a two-way ANOVA. *P < 0.05, ****P < 0.0001

Supplementary Figure 6

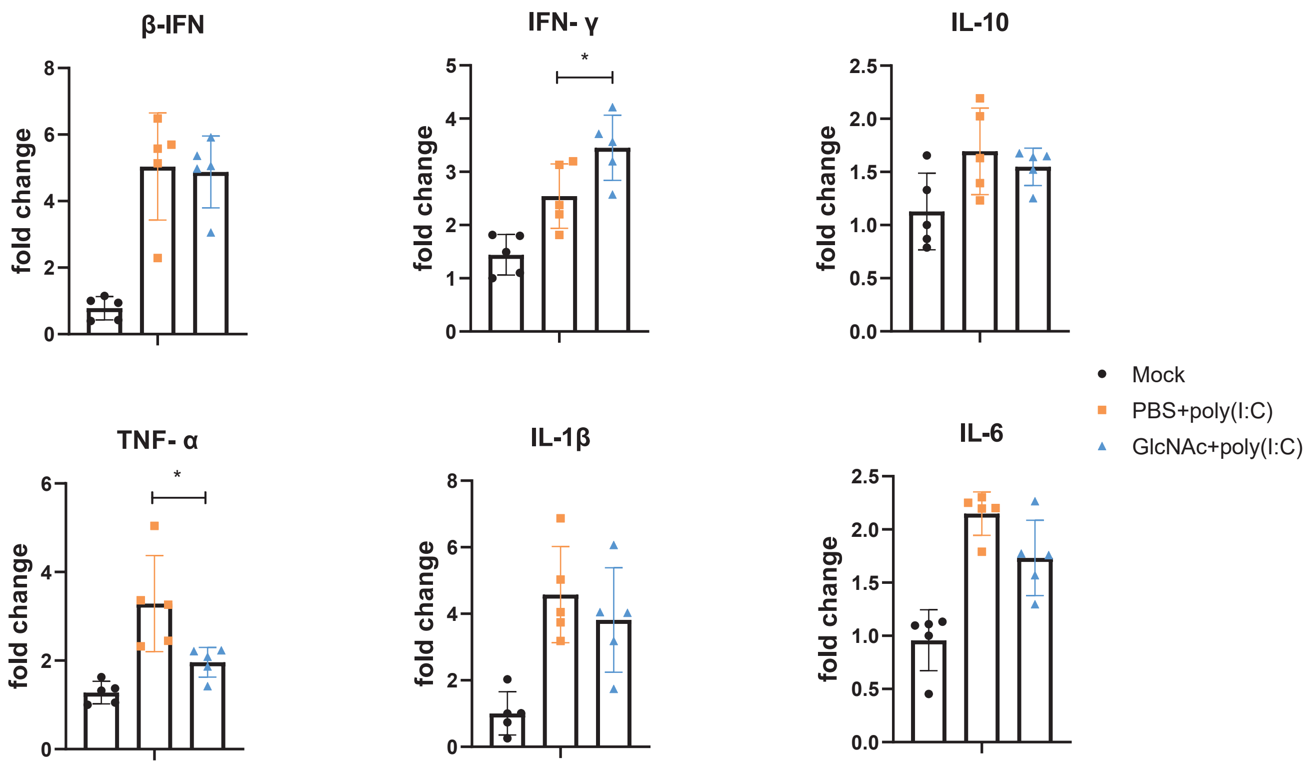

Fig. S6. The mRNA level of IFN-β, IFN-γ, IL-6, IL-1β, IL-10, and TNF-α in the lung were measured by qPCR. The data are shown as the means ± SD (n = 5, for each time point per group). *P < 0.05 via two-way ANOVA.

Supplementary Figure 7

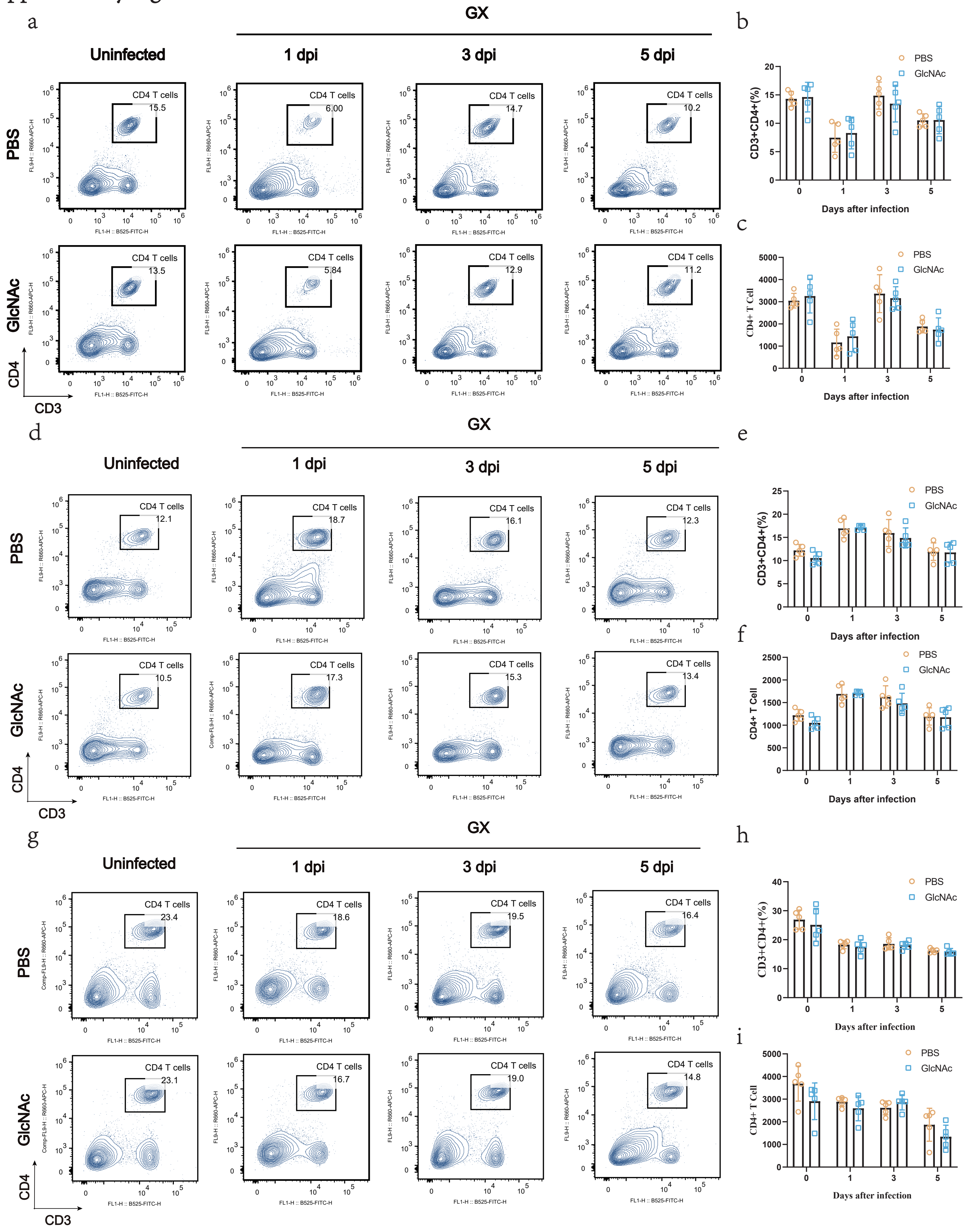

Fig. S7. Oral administration of GlcNAc did not affect the proportion of CD4+ T cells before and after infection in mice. The experimental scheme is as shown in Figure 5. (a, b and c) Lymphocytes from local draining mediastinal peripheral blood, (d, e and f) lung, (g, h and i) or spleen were analyzed by flow cytometry.Representative flow cytometry plots with gating strategy (a), summary graphs showing the proportion(b) and number(c) of CD4+ T cells in lymphocytes in peripheral blood post infection. (d, e and f) As in (a, b and c), but showing the lymphocytes from the lung. (g, h and i) As in (d, e and f), but showing the NK cells from the spleen. Statistical analysis data were assessed using a two-way ANOVA.

Supplementary Figure 8

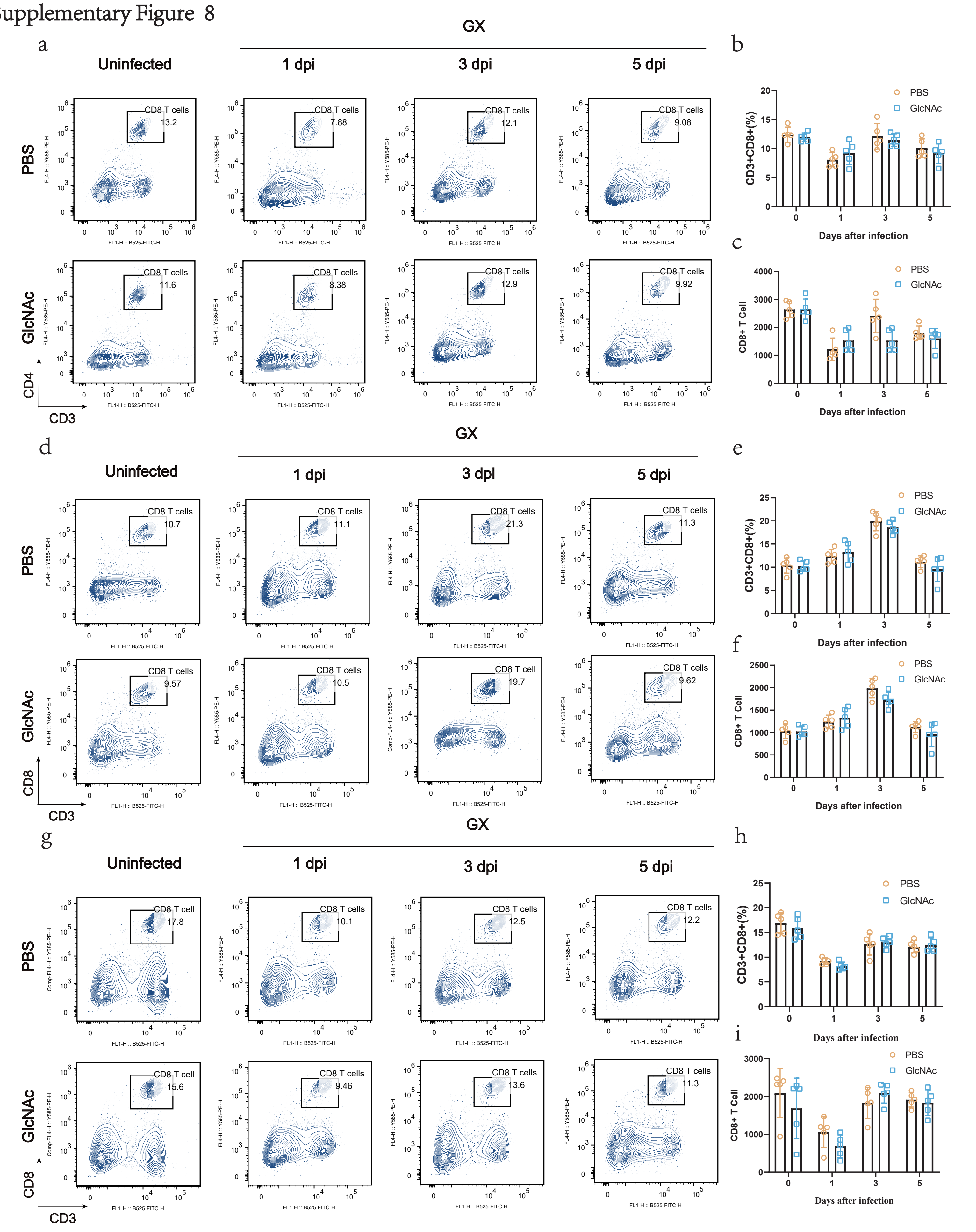

Fig. S8. Oral administration of GlcNAc did not affect the proportion of CD8+ T cells before and after infection in mice. The experimental scheme is as shown in Figure 5. (a, b and c) Lymphocytes from local draining mediastinal peripheral blood, (d, e and f) lung, (g, h and i) or spleen were analyzed by flow cytometry.Representative flow cytometry plots with gating strategy (a), summary graphs showing the proportion(b) and number(c) of CD8+ T cells in lymphocytes in peripheral blood post infection. (d, e and f) As in (a, b and c), but showing the lymphocytes from the lung. (g, h and i) As in (d, e and f), but showing the NK cells from the spleen. Statistical analysis data were assessed using a two-way ANOVA.

Supplementary Figure 9

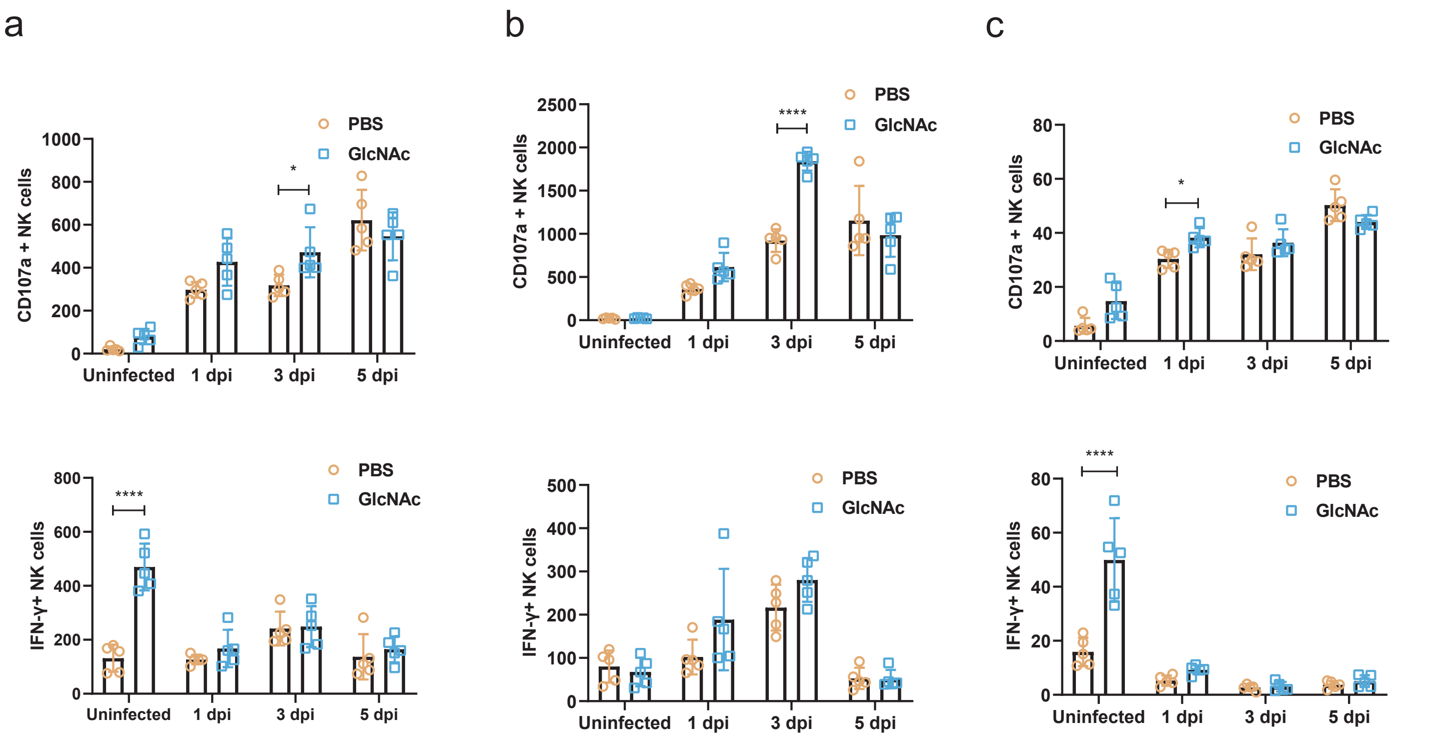

Fig.S9 . Number of IFN-γ and CD107a NK cells in blood (a), lung (b) and spleen(c) were assessed using a two-way ANOVA. *P < 0.05, ****P < 0.0001.

Supplementary Figure 10

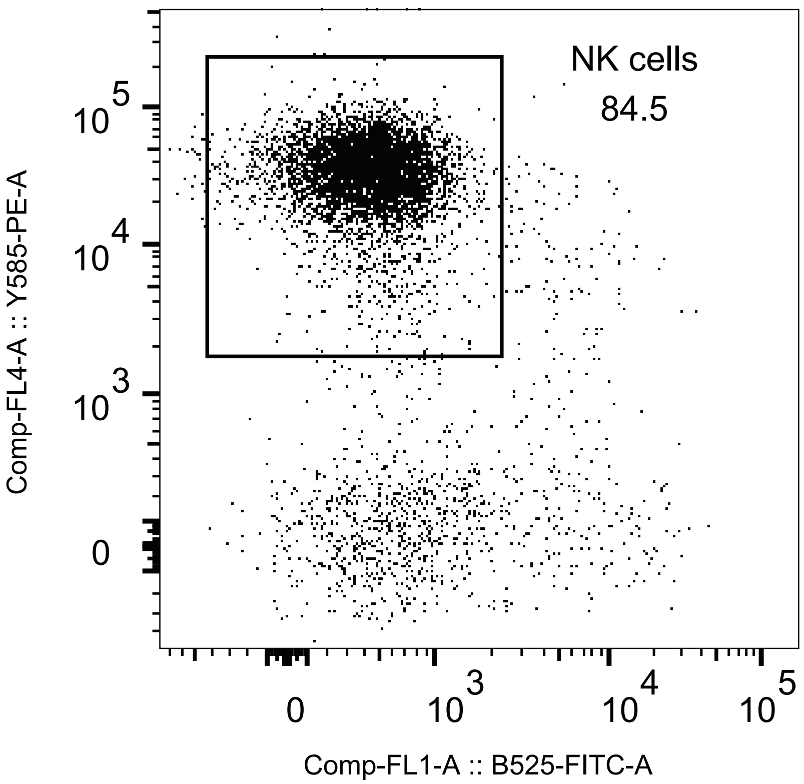

Fig. S10. Flow cytometric analysis confirmed that the purity of NK cells was greater than 80%. Experimental description references to Fig. 6. NK cells were purified using an EasySep Mouse NK Cell Isolation Kit according to the manufacturer's instructions and then the purity of NK cells was determined by flow cytometry.

Supplementary Figure 11

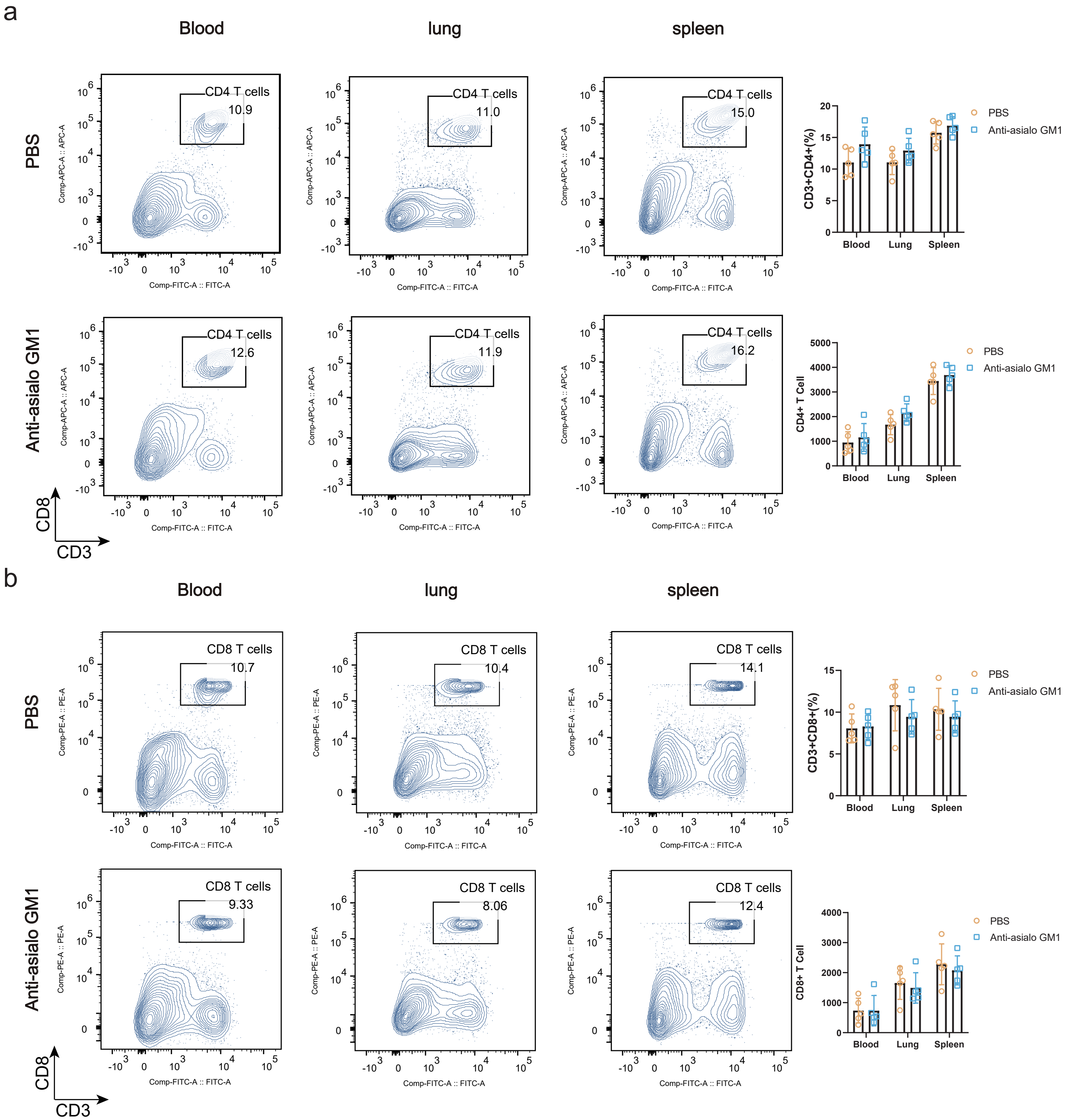

Fig. S11. Anti-asialo GM1 does not affect CD4+ and CD8+ T cells. Lymphocytes from local draining mediastinal peripheral blood, lung, or spleen were analyzed by flow cytometry.Representative flow cytometry plots with gating strategy, summary graphs showing the proportion and number of CD4+ T cells(a) and CD8+ T cells (b) in lymphocytes

Supplementary Figure 12

a

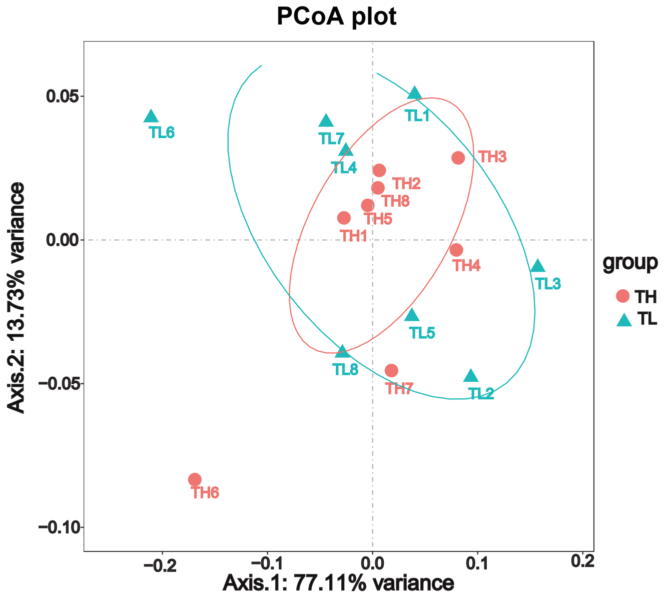

b

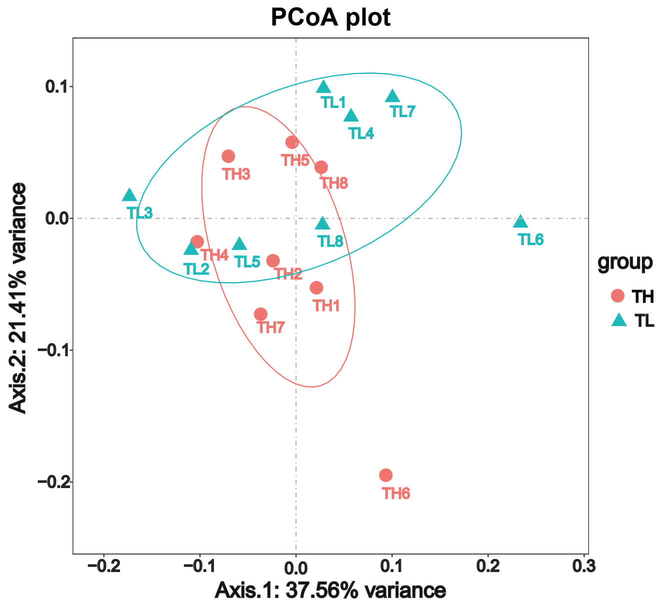

Fig. S12. PCoA of the weighted UniFrac distances between the TH and TL group at the phylum level (a) and genus level (b). Experimental description references to Fig. 7.

Supplementary Figure 13

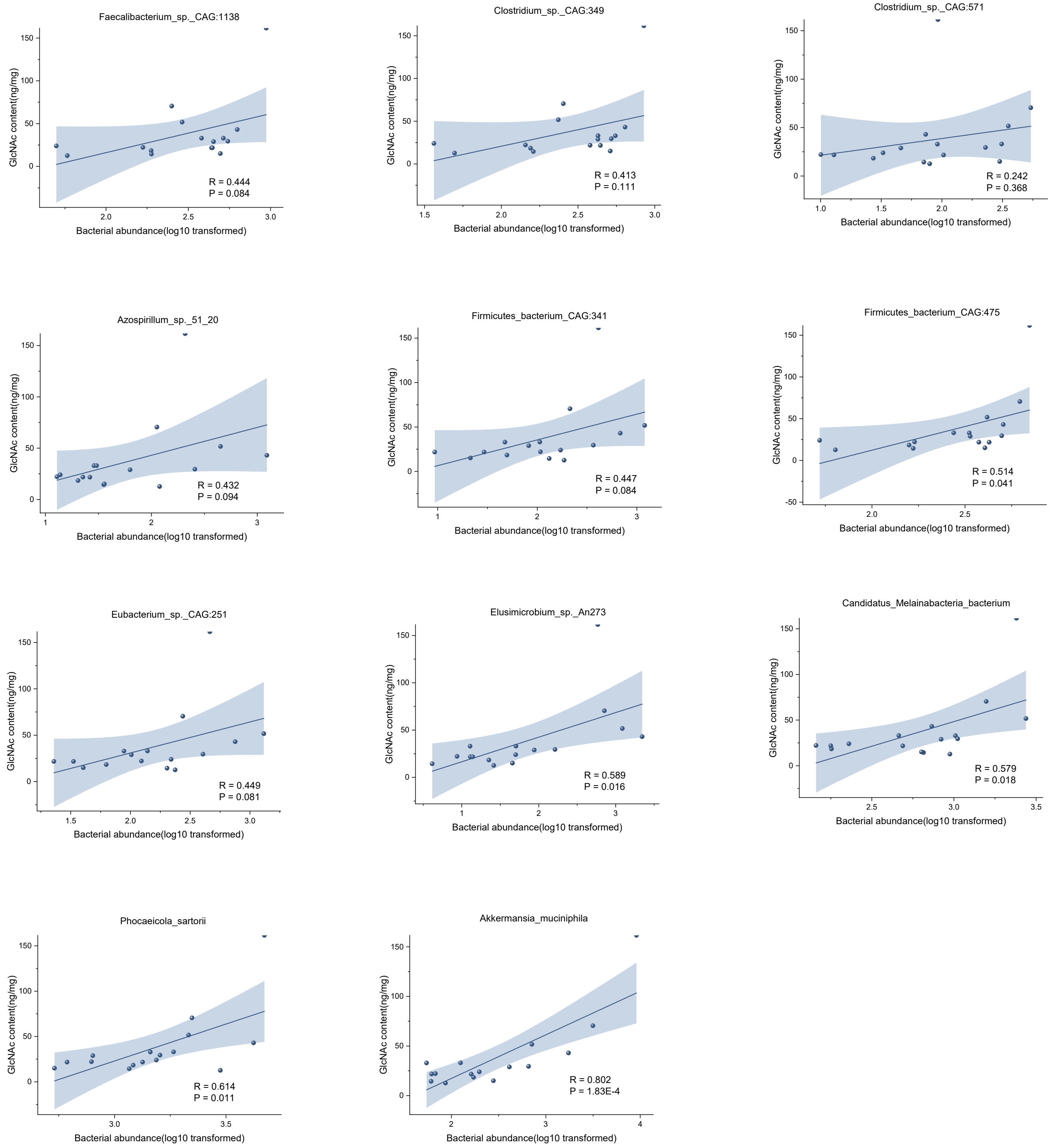

Fig. S13. Association of gut microbiota relative abundance and content of GlcNAc. Correlation between gut microbiota relative abundance and content of GlcNAc was examined using Spearman's correlation test. Regression lines with 95% CI (blue area) were shown on scatter plots.

Supplementary Figure 14

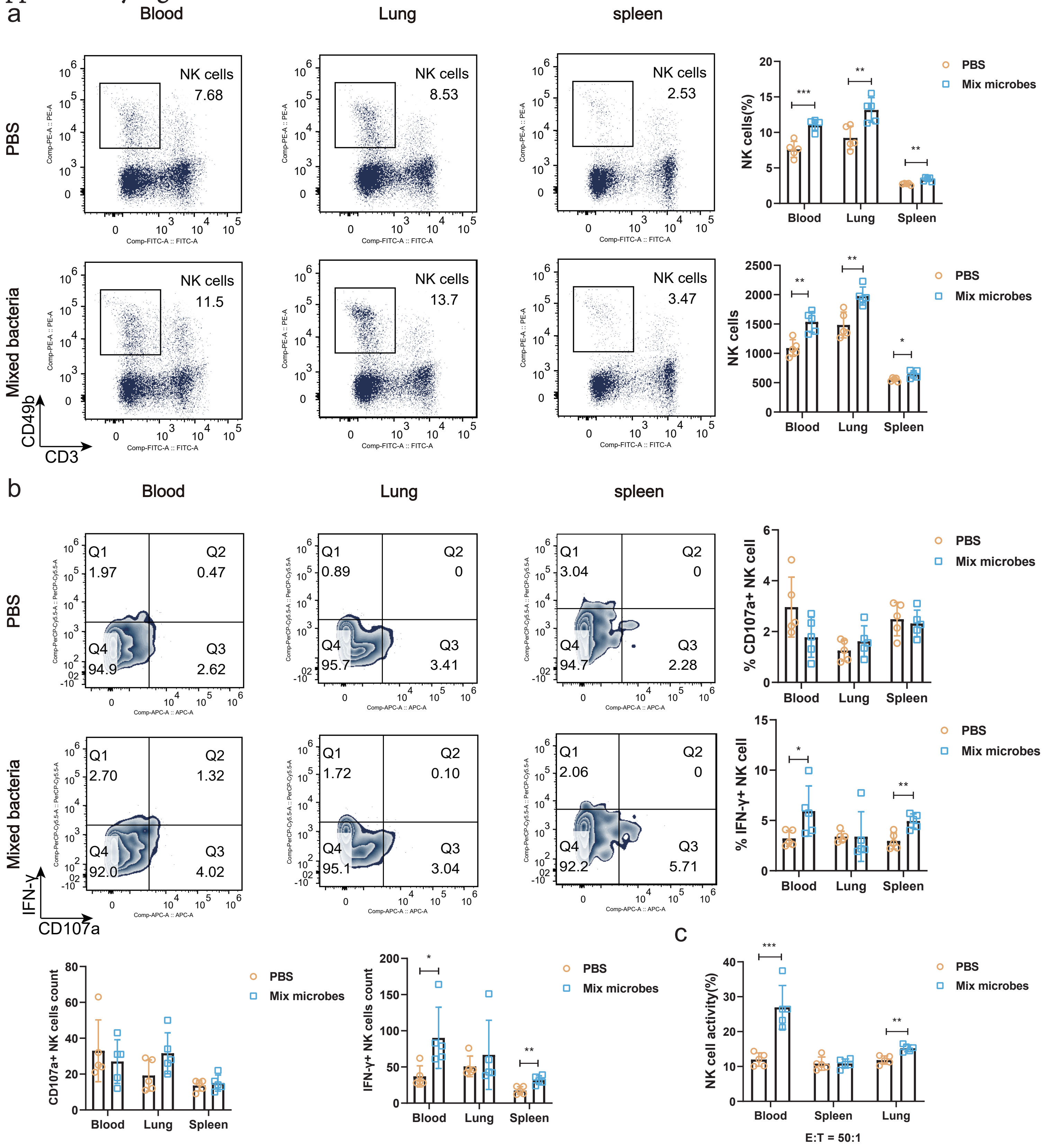

Fig. S14. Oral administration of mixed bacteria increases the proportion and activity of NK cells. SPF mice were orally administered either PBS or PBS containing 1×10^8 CFU of Clostridium sp., P. sartorii, and A. muciniphila daily for a duration of 1 week. Lymphocytes from local draining mediastinal peripheral blood, lung, or spleen were analyzed by flow cytometry. Representative flow cytometry plots with gating strategy and summary graphs showing the proportion of NK cells (a) in lymphocytes. (b)Frequency of IFN-γ and CD107a surface expression in gated CD3-CD49B+ NK cells. (c)NK cells were isolated from peripheral blood, lung, and spleen and their activities were measured by a calcein-release-assay. Statistical analysis data were assessed using a Student's t -test. * $p < 0.05$; **, $p < 0.01$; *** $p < 0.001$.

Supplementary Figure 15

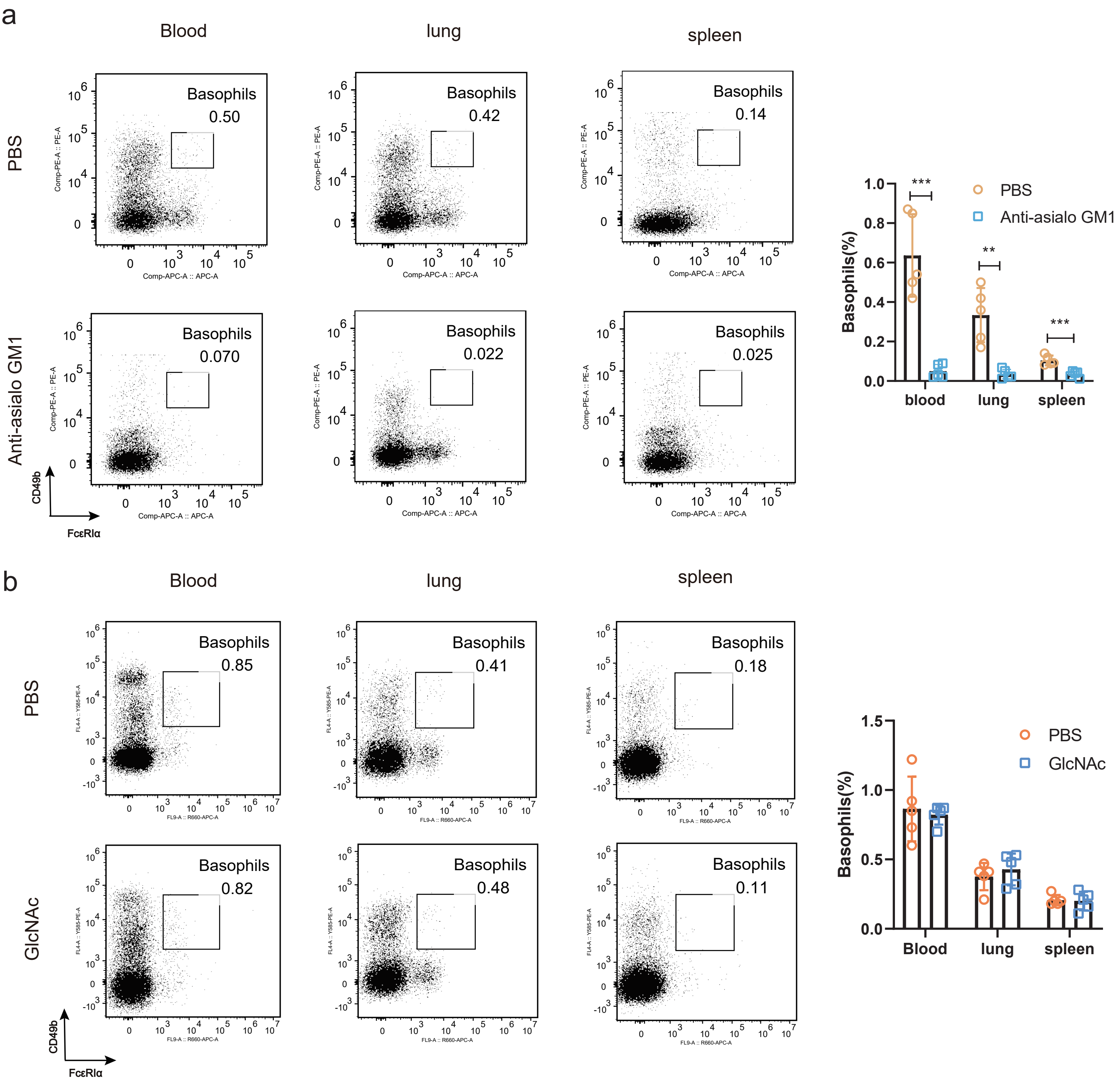

Fig. S15. The Basophils were determined by flow cytometry. (a)Basophils in the blood, lung, and spleen were analyzed by flow cytometry in mice following intraperitoneal injection of either PBS or anti-asialo GM1. (b)Flow cytometry was used to analyze basophils in the blood, lung, and spleen of mice after oral administration of PBS or GlcNAc. Statistical analysis was performed using the Student’s t -test analysis

Supplementary Figure 16

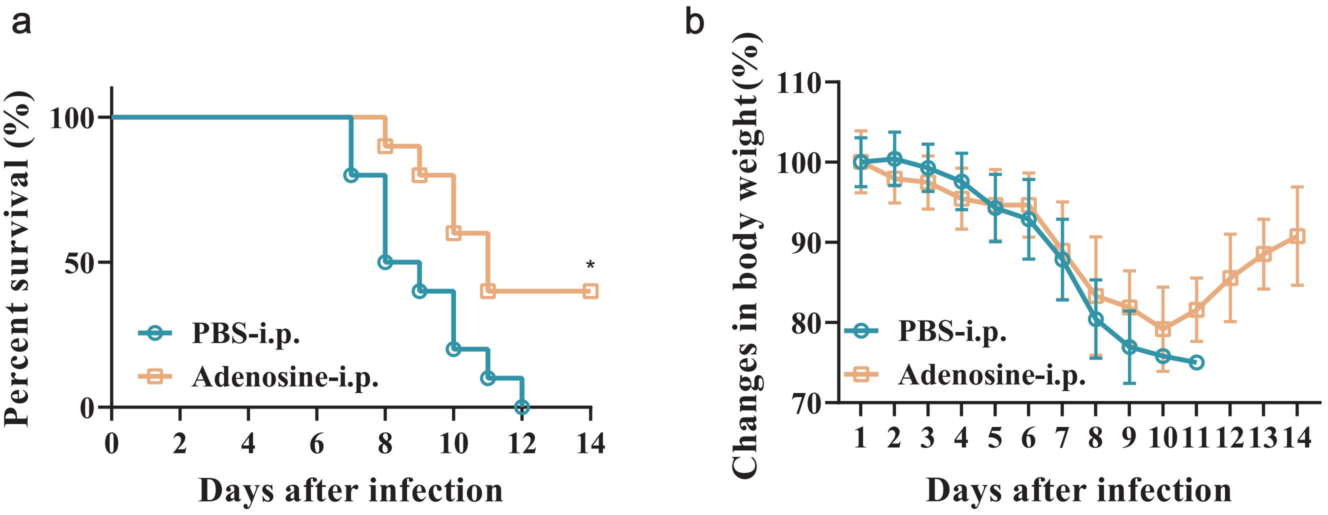

Fig. S16. Adenosine improves survival of influenza-infected mice by intraperitoneal injection. SPF mice were divided into PBS group and adenosine group (n = 10). Before infection, mice were injected intraperitoneally with 2 mg/kg adenosine or PBS every other day for 3 times. All mice were intranasally inoculated with 1 × 104 EID50 of the H7N9 influenza virus. At 1 and 3 dpi, mice of adenosine group were injected intraperitoneally with 2 mg/kg adenosine. The survival(a) and body weights(b) of the mice were monitored daily for 15 days (0–14 days post -infection).

Supplementary Material Methods

Detail methods of Metabolomics profiling

LC-MS untargeted metabolomics

60mg sample was transferred into a 2 mL tube, and add 200ul of water, vortex for 60s, then add 800ul of methanol-acetonitrile solution (1:1, v/v), and vortex for 60s. Ultrasonic at low temperature for 30 min, 2 times, and placed at -20 °C for 1 h to precipitate proteins. After centrifugation at 14,000 rcf for 20 min at 4 °C, the supernatant was freeze-dried and the samples were stored at -80 °C. The samples were separated by Agilent 1290 infinity LC (Agilent) Ultra High Performance Liquid Chromatography system (UHPLC) HILIC column; Column temperature 25 ℃; Flow rate 0.3 ml\/min; Mobile phase composition a: water +25 mM ammonium acetate +25 mm ammonia water, b: acetonitrile; The gradient elution procedure is as follows: 0-0.5 min, 95% B; 0.5---7min, B changes linearly from 95% to 65%; 7-8 min, B changes linearly from 65% to 40%; 8-9 min, B maintained at 40%; 9-9.1 min, B changes linearly from 40% to 95%; 9.1-12 min, B maintained at 95%; During the whole analysis process, the sample is placed in the 4 ℃ automatic sampler. In order to avoid the influence caused by the fluctuation of instrument detection signal, the random sequence is used for continuous analysis of samples. QC samples are inserted into the sample queue to monitor and evaluate the stability of the system and the reliability of experimental data. Electro spray ionization (ESI) positive and negative ion modes were used for detection. The samples were separated by UHPLC and analyzed by Agilent 6550 mass spectrometer (Agilent). ESI source conditions are as follows: gas tem: 250 ℃, drying gas: 16 L/min, nebulizer: 20 psig, sheath gas tem: 400 ℃, sheath gas flow: 12 L/min, vcap: 3000 V, nozzlevoltage: 0 V. fragment: 175 V, mass range: 50-1200, acquisition rate: 4 Hz, cycle time: 250 ms. After the sample is tested, the metabolites are identified by ab triple TOF 6600 mass spectrometer, and the primary and secondary spectra of QC samples are collected. ESI source conditions are as follows: ion source gas1 (gas1): 40, ion source gas2 (gas2): 80, curtain gas (cur): 30, source temperature:650 ℃, ionsapary voltage floating (isvf) ± 5000 V (positive and negative modes); The secondary mass spectrum is obtained by information dependent acquisition (IDA), and the high sensitivity mode is adopted. The declining potential (DP): ± 60 V (positive and negative modes), the collision energy: 35 ± 15 ev. The IDA settings are as follows: exclude isotopics within 4 Da,

candidate ions to monitor per cycle:10. Data acquisition is divided into sections according to mass range, 50-300, 290-600, 590-900, 890-1200, so as to expand the acquisition rate of secondary spectrogram. Each method collects four repetitions in each section. The collected data were used to identify the structure of metabolites by MetDDA and LipDDA methods respectively. The original data is converted into MzXML format, and then use XCMS program for peak alignment, retention time correction and peak area extraction. The structure of metabolites was identified by means of accurate mass number matching (<25ppm) and secondary spectrum matching, and database was retrieved. For the data extracted by xcms, delete the ion peak with the group sum > 2/3. After the data is preprocessed by Pareto scaling, multidimensional statistical analysis is carried out.

GC-MS untargeted metabolomics

50±1 mg sample was transferred into a 2 mL tube, and 500 μL pre-cold extraction mixture (methanol/chloroform (v:v) =3:1) with 10 μL internal standard (adonitol, 0.5 mg/mL stock) were added. Samples were vortexed for 30 s and homogenized with ball mill for 4 min at 35 Hz, followed by ultrasonication for 5 min in ice water. After centrifugation at 4 °C for 15 min at 12000 rpm, 200 μL supernatant was transferred to a fresh tube. To prepare the QC (Quality control) sample, 50 μL of each sample was taken out and combined together.After evaporation in a vacuum concentrator, 40 μL of Methoxyamination hydrochloride (20 mg/mL in pyridine) was added and then incubated at 80 °C for 30 min, then derivatized by 60 μL of BSTFA regent (1% TMCS, v/v) at 70 °C for 1.5 h. Gradually cooling samples to room temperature, 5 μL of FAMEs (in chloroform) was added to QC sample. All samples were then analyzed by gas chromatograph coupled with a time-of-flight mass spectrometer (GC-TOF-MS). GC-TOF-MS analysis was performed using an Agilent 7890 gas chromatograph coupled with a time-of-flight mass spectrometer. The system utilized a DB-5MS capillary column. 1 μL aliquot of sample was injected in splitless mode. Helium was used as the carrier gas, the front inlet purge flow was 3 mL min−1, and the gas flow rate through the column was 1 mL min−1. The initial temperature was kept at 50 °C for 1 min, then raised to 310 °C at a rate of 10 °C min−1, then kept for 8 min at 310 °C. The injection, transfer line, and ion source temperatures were 280, 280and 250 °C, respectively. The energy was -70 eV in electron impact mode. The mass spectrometry data were acquired in full-scan mode with the m/z range of 50-500 at a rate of 12.5 spectra per second after a solvent delay of 6.25 min.A total of 722 peaks

were detected in this experiment. Raw data analysis, including peak extraction, baseline adjustment, deconvolution, alignment and integration, was finished with Chroma TOF (V 4.3x，LECO) software[1] and LECO-Fiehn Rtx5 database was used for metabolite identification by matching the mass spectrum and retention index. Finally, the peaks detected in less than half of QC samples or RSD＞30% in QC samples was removed[2].

Determining viral loads in the lungs of mice treated with and without antibiotics after influenza infection

20 SPF mice were randomly divided into two groups (n = 10), o antibiotic treatment group and normal group. Antibiotic treatment was as previously described. All mice were intranasally infected with influenza virus under anesthesia as previously described. The lungs were collected on the fifth day of infection, and the copy number of lung vRNA was detected by QPCR. The pCAGGS-NP plasmid was used to generate a standard curve with which the number of vRNA were calculated.

The mRNA levels of Cytokine in lung detected by qPCR after poly(IC) stimulation.

15 mice were randomly divided into three groups (n = 5): MOCK, PBS+poly(IC), and GlcNAc+poly(IC) groups. The groups PBS+poly(IC) and GlcNAc+poly(IC) were administered orally with 200ul of PBS or 1000 mg/kg GlcNAc, respectively, while the Mock group was not treated. Mice in the PBS group and GlcNAc group were anesthetized with ketamine and inoculated with 50 µg poly(I:C) through nasal drops for 2 consecutive days and then lung samples are collected. The mRNA levels of cytokine in lung homogenates were assessed

Determining basophils responses by flow cytometry

SPF mice were randomly assigned to two groups (n = 5): a PBS and GlcNAc group. Mice in the PBS group were orally administered 200 µL PBS, and those in the GlcNAc group were administered 1000 mg/kg GlcNAc per day for a week. Influenza infection was performed as described above. The blood, spleen, and lungs of three mice from each group were collected to determine basophils responses by flow cytometry

Determination of GlcNAc content in *Clostridium sp., P. sartorii,* and *A.*

***muciniphila* culture supernatant by *UHPLC–MRM-MS*.**

Clostridium sp., P. sartorii, and *A. muciniphila* were cultured anaerobically for 48 hours with 3 replicates per group. The bacterial solution was then centrifuged at 4000 rpm for 5 minutes, and the supernatants were collected. These supernatants were passed through a 0.22um filter membrane and stored at -80 degrees Celsius for future use. The GlcNAc content in the samples was determined by UHPLC–MRM-MS.

The effect of anti-asialo GM1 on CD4+ T cells, CD8+ T cells and basophils was detected by flow cytometry

10 SPF mice were randomly assigned to two groups (n = 5): a PBS and GlcNAc group. Mice in the PBS group were injected with 100 μL PBS by intraperitoneal injection, and those in the GlcNAc group were treated with 50 μL anti-asialo GM1. The blood, spleen, and lungs of three mice from each group were collected to determine T cells and basophils responses by flow cytometry.

Effect of *Clostridium sp., P. sartorii,* and *A. muciniphila* on NK Cells

20 mice were randomly divided into two groups (n = 10): the PBS group and the mixed bacteria group. The mice in the PBS group were orally administered 200 ul of PBS per day, while the mice in the mixed bacteria group were orally administered 200 ul of PBS containing 1×10^9 CFU of *Clostridium sp., P. sartorii*, and *A. muciniphila* for 1 week. The blood, spleen, and lungs of 5 mice from each group were collected to determine NK cells responses by flow cytometry. Blood, spleen and lungs were collected from the remaining mice, NK cells were isolated using an NK cell sorting kit, and NK cell activity was assessed by a calcein release assay.

1. Kind, T. *et al.* FiehnLib: mass spectral and retention index libraries for metabolomics based on quadrupole and time-of-flight gas chromatography/mass spectrometry. *Analytical chemistry* **81**, 10038-10048 (2009).

2. Dunn, W.B. *et al.* Procedures for large-scale metabolic profiling of serum and plasma using gas chromatography and liquid chromatography coupled to mass spectrometry. *Nature protocols* **6**, 1060-1083 (2011).

Table S1. Reverse transcription primers

Reverse Primer	(5' to 3')
Uni12 primer	AGCAAAAGCAGG

Table S2. The primers used in qPCR.

Gene	Forward primer	Reverse primer
NP	AACGACCGGAATTTCTGGAGAGG	CCGTACACACAAGCAGGCAAGC

Table. S3. Liquid chromatographic gradient

Time (min)	Solvent A	Solvent B	Flow (μL/min)
0.0	80%	20%	300
2.5	80%	20%	300
3.0	5%	95%	300
7.0	5%	95%	300
7.2	80%	20%	300
10.0	80%	20%	300

Table S4 Target compound MRM

Analyte	Precursor	Product	CE/V	Ret Time (min)	Polarity
GlcNAc	222.0	138	15	2.025	Positive

Table. S5. Lung viral load of mice in TH group and TL group.

group		lung viral load（-log EID50/mL）
TH group	TH-1	5.41
	TH-2	5.375
	TH-3	5.5
	TH-4	5.625
	TH-5	5.68
	TH-6	5.41
	TH-7	5.4
	TH-8	5.375
TL group	TL-1	5.125
	TL-2	5.17 5
	TL-3	5
	TL-4	4.67
	TL-5	4.83
	TL-6	4.24
	TL-7	5
	TL-8	4.625

Table. S6. Differential metabolites between TH group and TL group (Display with excel file)

Table S7. Phenotype (viral laod)-associated metabolites in cecal contents

Metabolites	lung viral load	
	correlation coefficient (R)	P value
Adenosine	-0.558	0.025
GlcNAc	-0.538	0.032
cellobiose	-0.27	0.311
Citraconic acid	-0.386	0.14
Glu-Pro	-0.546	0.029
L-Arginine	-0.436	0.091
malonic acid	-0.238	0.375
maltose	-0.573	0.02

Table. S8. The PCoA of the weighted UniFrac distances between the TH and TL group at the genus level (Display with excel file.)

Table. S9. Comparison of the relative abundances of the major bacterial phyla(top10) representing the gut microbiota between the TH and TL group (Display with excel file.)

Table. S10. Comparison of the relative abundances of the major bacterial genus(top30) representing the gut microbiota between the TH and TL group (Display with excel file.)

Table. S11. The top 50 representative species with significant difference between TH group and TL group (Display with excel file.)

Table. S12. Taxa identified by LEfSe analysis in this study (Display with excel file

Table. S13. GlcNAc concentration of cecal contents in TH and TL group were determined by UHPLC–MRM-MS

Sample Name	Sample Weight (mg)	Extraction Volume (μL)	Dilution Factor	GlcNAc Metabolite Concentration (ng/mg)
TH-1	63.5	1000	10	23.97
TH-2	92.8	1000	10	18.37
TH-3	70.9	1000	10	14.42
TH-4	94.6	1000	10	15.03
TH-5	82.5	1000	10	21.70
TH-6	52.6	1000	10	12.63
TH-7	79.2	1000	10	21.77
TH-8	74.2	1000	10	22.15
TL-1	54.6	1000	10	28.89
TL-2	111	1000	10	32.99
TL-3	87.5	1000	10	32.91
TL-4	56.9	1000	10	70.47
TL-5	78.8	1000	10	161.57
TL-6	47.5	1000	10	43.03
TL-7	55.3	1000	10	51.68
TL-8	72.8	1000	10	29.45

The result is the detected concentration of the target compound (Metabolite Concentration), in ng/mg; the calculation formula is: Metabolite Concentration = Calculated Concentration * Dilution Factor * Relative Molecular Mass (MW) * Extract Volume (Extraction Volume)/sample mass; the result is the concentration of the target compound contained in the sample per mg.

Table S14. Phenotype (GlcNAc content)-associated microbes in cecal contents

microbes	lung viral load	
	correlation coefficient (R)	P value
Faecalibacterium_sp._CAG:1138	0.444	0.084
Clostridium_sp._CAG:349	0.413	0.111
Clostridium_sp._CAG:571	0.242	0.368
Azospirillum_sp._51_20	0.432	0.094
Firmicutes_bacterium_CAG:341	0.447	0.084
Firmicutes_bacterium_CAG:475	0.514	0.041
Eubacterium_sp._CAG:251	0.449	0.081
Elusimicrobium_sp._An273	0.589	0.016
Candidatus_Melainabacteria_bacterium	0.579	0.018
Phocaeicola_sartorii	0.614	0.011
Akkermansia_muciniphila	0.802	0.000183

Table. S15. GlcNAc concentration of cecal contents in bacterial culture supernatants were determined by UHPLC–MRM-MS

Sample Name	GlcNAc
	Calculated Concentration (ug/L)
Medium (*A. muciniphila*)-1	3.35
Medium (*A. muciniphila*)-2	4.20
Medium (*A. muciniphila*)-3	4.74
A. muciniphila-1	8.65
A. muciniphila-2	8.40
A. muciniphila-3	8.28
Medium (*P. sartorii*)-1	4.65
Medium (*P. sartorii*)-2	4.73
Medium (*P. sartorii*)-3	4.76
P. sartorii-1	2.77
P. sartorii-2	2.57
P. sartorii-3	2.84
Medium (*Clostridium sp*)-1	17.82
Medium (*Clostridium sp*)-2	17.28
Medium (*Clostridium sp*)-3	16.61
Clostridium sp-1	50.92
Clostridium sp-2	55.72
Clostridium sp-3	54.58

Supplementary Figure 16

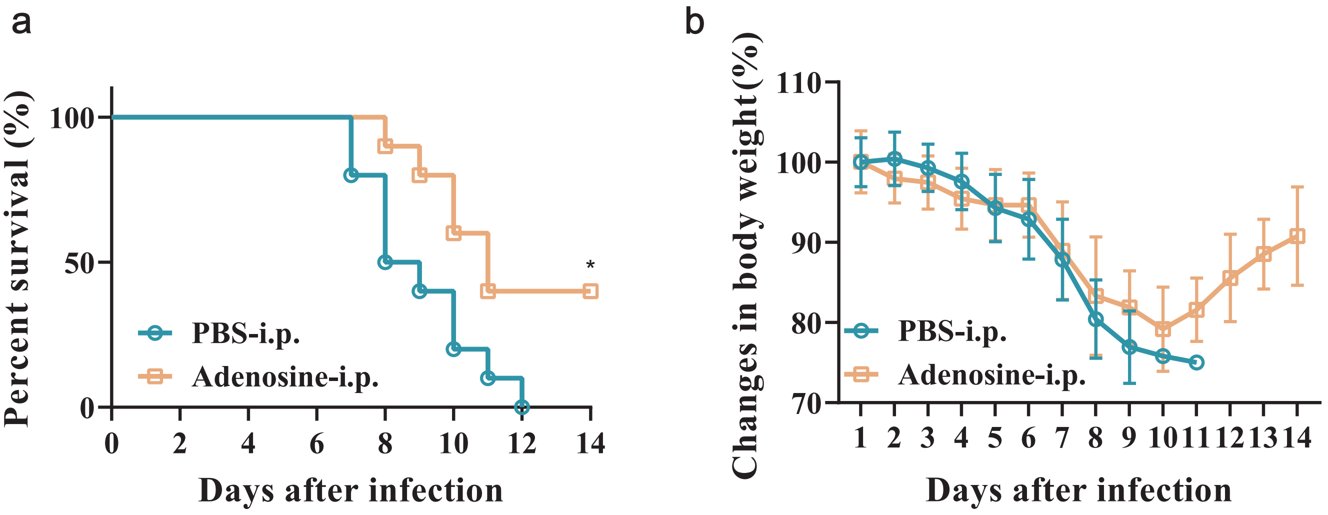

Fig. S16. Adenosine improves survival of influenza-infected mice by intraperitoneal injection. SPF mice were divided into PBS group and adenosine group (n = 10). Before infection, mice were injected intraperitoneally with 2 mg/kg adenosine or PBS every other day for 3 times. All mice were intranasally inoculated with 1 × 104 EID50 of the H7N9 influenza virus. At 1 and 3 dpi, mice of adenosine group were injected intraperitoneally with 2 mg/kg adenosine. The survival(a) and body weights(b) of the mice were monitored daily for 15 days (0–14 days post -infection).
